# Biomolecules Turn Self-Assembling Amphiphilic Block Co-polymer Platforms Into Biomimetic Interfaces

**DOI:** 10.3389/fchem.2018.00645

**Published:** 2019-01-08

**Authors:** Saziye Yorulmaz Avsar, Myrto Kyropoulou, Stefano Di Leone, Cora-Ann Schoenenberger, Wolfgang P. Meier, Cornelia G. Palivan

**Affiliations:** Department of Chemistry, University of Basel, Basel, Switzerland

**Keywords:** self-assembly, amphiphilic block copolymers, micelles, polymersomes, supported polymer membranes, biomolecules conjugation, biomedical applications

## Abstract

Biological membranes constitute an interface between cells and their surroundings and form distinct compartments within the cell. They also host a variety of biomolecules that carry out vital functions including selective transport, signal transduction and cell-cell communication. Due to the vast complexity and versatility of the different membranes, there is a critical need for simplified and specific model membrane platforms to explore the behaviors of individual biomolecules while preserving their intrinsic function. Information obtained from model membrane platforms should make invaluable contributions to current and emerging technologies in biotechnology, nanotechnology and medicine. Amphiphilic block co-polymers are ideal building blocks to create model membrane platforms with enhanced stability and robustness. They form various supramolecular assemblies, ranging from three-dimensional structures (e.g., micelles, nanoparticles, or vesicles) in aqueous solution to planar polymer membranes on solid supports (e.g., polymer cushioned/tethered membranes,) and membrane-like polymer brushes. Furthermore, polymer micelles and polymersomes can also be immobilized on solid supports to take advantage of a wide range of surface sensitive analytical tools. In this review article, we focus on self-assembled amphiphilic block copolymer platforms that are hosting biomolecules. We present different strategies for harnessing polymer platforms with biomolecules either by integrating proteins or peptides into assemblies or by attaching proteins or DNA to their surface. We will discuss how to obtain synthetic structures on solid supports and their characterization using different surface sensitive analytical tools. Finally, we highlight present and future perspectives of polymer micelles and polymersomes for biomedical applications and those of solid-supported polymer membranes for biosensing.

## Introduction

Biological membranes are of great importance in life as they play crucial roles in the structure and function of all living cells. They serve as an interface between cells and their environment and provide the basis for internal compartmentalization which is essential for controlling many cellular processes. Membranes also take part in distinct biological processes including selective nutrient transport, signal transduction, cell-cell recognition, and inter-and intra-cellular communication. Structurally, they consist of many different components, mainly distinct phospholipids and cell-specific sets of proteins. While the phospholipid bilayer provides the structural backbone of the membrane, proteins (e.g., peripheral and transmembrane proteins) are incorporated in or attached to the phospholipid matrix (Singer and Nicolson, 1972). In the past few decades, much attention has been devoted to developing novel model biointerfaces that mimic basic functions of biological membranes.

**Figure 1:**
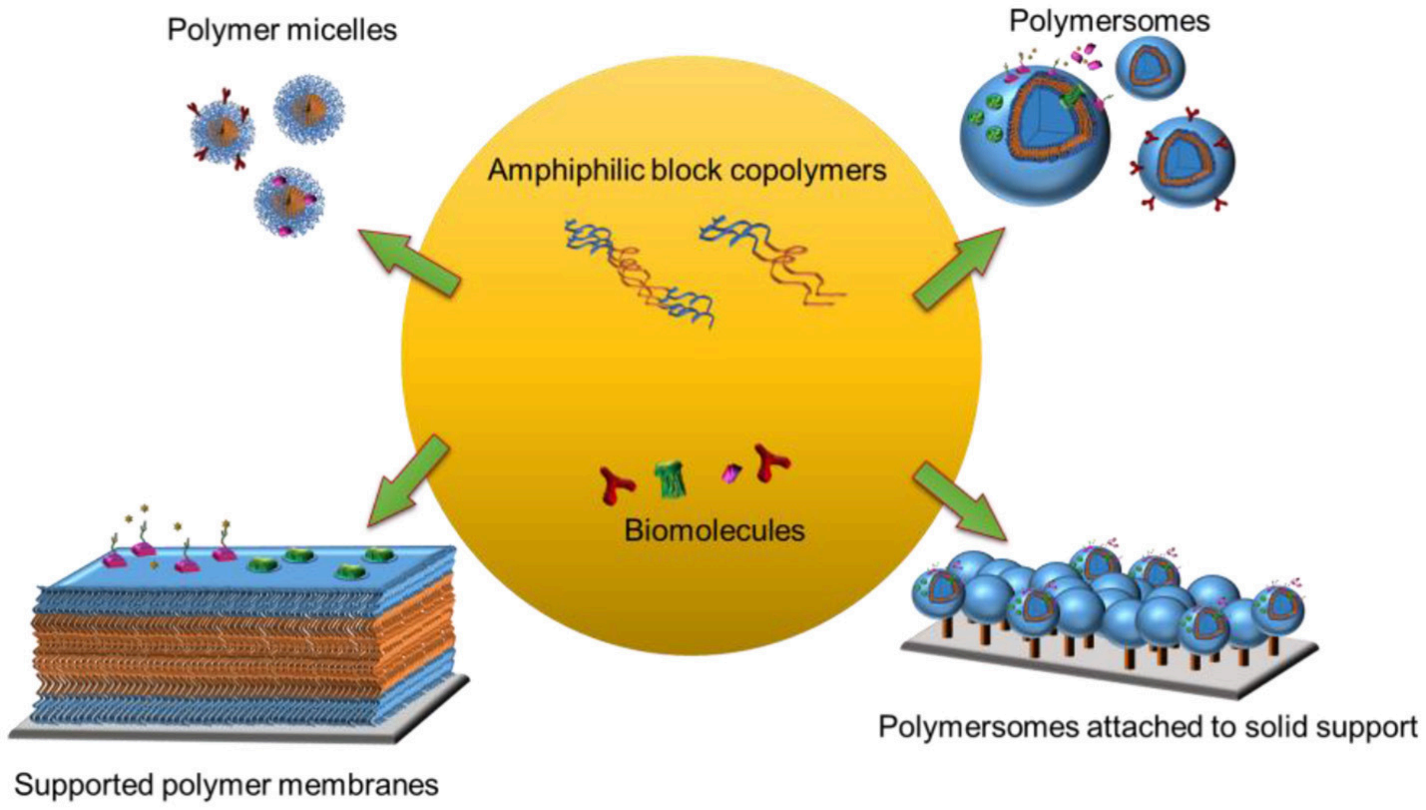
Biomolecules turn block co-polymer platforms into functional biointerfaces.

In a biomimetic approach, naturally occurring lipids, synthetic lipids, and synthetic block copolymers have been used to create the membrane backbone (Sackmann, 1996; Mecke et al., 2006). Polymer-derived synthetic model membrane systems have several advantages over those made of phospholipids since they are more stable and more amenable to chemical modifications (Discher et al., 1999; Discher and Ahmed, 2006; Egli et al., 2011). To date, a range of simplified model systems including polymer micelles (Kulthe et al., 2012) and polymersomes (Discher and Ahmed, 2006), and polymersomes or planar membranes attached to solid supports have been obtained (Belegrinou et al., 2011; Rein et al., 2016) (Figure 1). Moreover, an increased complexity of the synthetic membranes, such as stimuli responsiveness (Li and Keller, 2009) or permeability (Battaglia et al., 2006) can be achieved by mixing block copolymers with different physicochemical properties and chemical modifications. Biological functions are brought about by combining the synthetic membranes with a variety of biomolecules including proteins, peptides, and nucleic acids. These biomolecules can be conjugated, inserted and encapsulated within three-dimensional polymer assemblies, whereas they can be conjugated to or inserted into planar polymer membranes.

In this review, we describe the self-assembly of block copolymers into micelles and vesicles in aqueous solution. We selected to focus on nanometer-range vesicles, so called polymersomes, while details related to vesicles in the micrometer range, so called giant unilamellar vesicles (GUVs) are reviewed elsewhere (Howse et al., 2009). The basic criteria underlying the self-assembly of amphiphilic block copolymers are introduced, however, without considering the full complexity of the process. We discuss how to prepare polymer micelles and polymersomes by the most commonly used methods, and how to equip them with different biomolecules. We then present procedures to immobilize polymersomes with and without biomolecules on solid supports and expand on selected functions obtained by specific biomolecules. We describe how to prepare planar polymer membranes on a solid support and how these platforms turn into biomimetic interfaces by attaching or inserting biomolecules to the polymer membranes. We conclude with touching on present and future perspectives of hybrid bio-polymer nanosystems in biomedical applications.

## Criteria for Self-Assembly of Amphiphilic Block Copolymers

**Figure 2:**
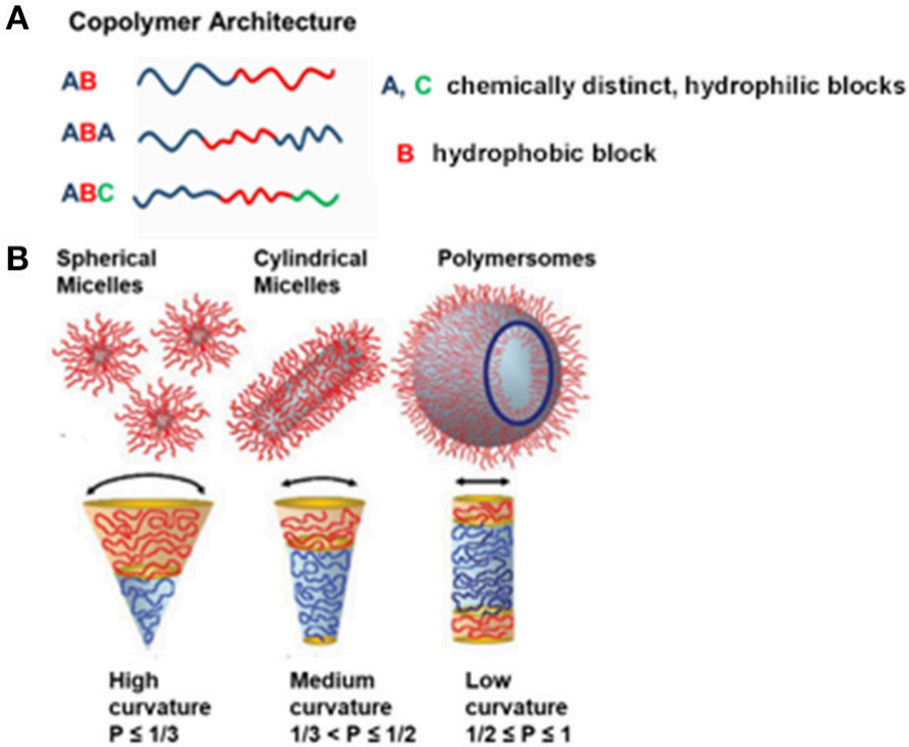
**(A)** Illustration representing different obtainable block copolymer architectures from different synthesis methods, and **(B)** various self-assemblies from amphiphilic block copolymers in an aqueous solution, depending on the packing parameter, p. Reproduced (with adaptations) from Blanazs et al. (2009) and Beales et al. (2017) with permission of Copyright © 2009 WILEY-VCH Verlag GmbH & Co. KGaA, Weinheim and Copyright © 2017 The Author(s) Paul A. Beales, respectively.

Advances in polymer chemistry have brought about many different strategies for producing amphiphilic block copolymers with desired numbers (n) and types (m) of monomers, which each have distinct hydrophilic and hydrophobic characteristics. To date, amphiphilic block copolymers are synthesized through different approaches including atom transfer radical polymerization (ATRP), reversible addition-fragmentation chain transfer (RAFT), or nitroxide mediated polymerization (NMP) (Matyjaszewski and Spanswick, 2005; Feng et al., 2017; Guo X. et al., 2018; Konishcheva et al., 2018). For example, synthesis of two chemically distinct monomers (m = 2) leads to a diblock copolymer (n = 2) whereas synthesis of three different monomers (m = 2 if two of the monomers are chemically same or m = 3 if the monomers are chemically distinct) results in triblock copolymers (n = 3). In general, these block copolymers are defined as AB diblock copolymers or ABA and ABC triblock copolymers where A and C are chemically distinct hydrophilic blocks whereas B represents the hydrophobic block (Figure 2A). Varying the number of blocks (n) and chemically distinct block types (m) in polymer synthesis creates a collection of unique copolymers, where each generates a specific nano-sized structure in aqueous solution. There are primary and secondary factors that change the self-assembly properties of block copolymers in the solution. The main primary factors are number of monomers (n), number of monomer types (m), degree of polymerization of each monomer (N_i_), and the associated interaction parameters (X_ij_), where i and j correspond to chemically distinct repeat units. Secondary factors include block flexibility (e.g., stiff vs. flexible chains), dispersity ####(*Đ*) and heterogeneity in block composition. A detailed discussion on these factors can be found elsewhere (Bates et al., 2012). In particular, ####*Đ* is influenced by the ratio of weight average (Mw) to number average (Mn) molecular weight (Lynd and Hillmyer, 2005), simply reflecting the molecular weight distribution. For example, the poly(ethylene oxide)-block-polycaprolactone (PEO-PCL) block copolymer with a polydispersity index of 1.14 yields predominantly polymersomes (Qi et al., 2013) whereas the PEO-PCL block copolymer with a polydispersity of 1.42 creates mainly worms (Rajagopal et al., 2010).

Amphiphilic block copolymers are able to self-assemble a wide range of nano-sized structures in aqueous solution. The most prominent examples are nanoparticles, micelles (spherical, cylindrical, and worm-like), and polymersomes. It is important to note that obtaining different block copolymer assemblies is the result of the inherent molecular curvature arising from the relative size difference between the hydrophilic and hydrophobic blocks. This principally defines the geometric packing of block copolymers in the resulting copolymer assemblies in aqueous solution, which is known as dimensionless packing parameter, p (Figure 2B). The p is defined as p = v/a_o_l_c_, where v is the volume of the hydrophobic block, a_o_ is the contact area of head group and l_c_ is length of hydrophobic block (Israelachvili, 2011). Although p has originally been developed for amphiphiles in water (Israelachvili et al., 1976), it has also been used for predicting structures resulting from self-assembly of amphiphilic block copolymers in aqueous solution (Kita-Tokarczyk et al., 2005). It corresponds to the ratio of the molecular volume of the hydrophobic block to the actual volume occupied by the block copolymer in the resulting assemblies. Depending on the p value, different polymer structures within aqueous solution have been predicted. For example, spherical micelles are formed when *p* ≤ 1/3, cylindrical micelles are formed when 1/3 < *p* ≤ 1/2, and vesicles are formed when 1/2 ≤ *p* ≤ 1 (Figure 2B) (Smart et al., 2008; Blanazs et al., 2009). In addition to primary and secondary factors which focus on the nature of block copolymers, external factors such as temperature, ionic strength, and pH also influence the self-assembly of particular block copolymers (Bae et al., 2003; Solomatin et al., 2003; Park et al., 2007).

Once desired block copolymer assemblies in an aqueous solution are obtained, further characterization is required for these assemblies to qualify for specific biomedical and biosensing applications. The characterization of the assemblies by a wide range of microscopy and light scattering techniques mostly concerns size, size distribution, shape, and surface charge. For example, optical microscopy has been mainly used for polymersomes that are bigger than limit of resolution (>200 nm) whereas transmission electron microscopy (TEM) has been utilized for high resolution images of both micelles and polymersomes (Habel et al., 2015; Men et al., 2016; Ruiz-Pérez et al., 2016). Static light scattering (SLS) and dynamic light scattering (DLS) have been employed to obtain radius of gyration (R_g_) and hydrodynamic radius (R_h_) of both micelles and polymersomes, respectively (Stauch et al., 2002). Specifically, if the ratio of R_g_ to R_h_, or shape factor, is ≤ 1, it is indicative of spherical objects (Brewer and Striegel, 2011) allowing for a prediction of the predominant morphology of assemblies in a solution. For example, polymersomes assembled from poly(ethylene glycol)-*b*-poly(D,L-lactide) (PEG-PDLLA) had a ratio close to 1, whereas the PEG-PDLLA elongated tubes had an average shape factor of 1.5 with all values above 1.3 (Abdelmohsen et al., 2016). In addition, DLS measurements will reveal the size distribution (e.g., unimodal, bimodal, or multimodal) of the assemblies (Habel et al., 2015). Apart from size parameters, zeta potential measurements can be carried out to determine the surface charge of assemblies (Hu et al., 2017).

## Polymer Micelles

Copolymer micelles are nano-sized assemblies formed by amphiphilic block copolymers in dilute solution when the concentration of a polymer is above the critical micellar concentration (CMC). The main two types of polymer micelles in aqueous solution are spherical and cylindrical micelles. Spherical micelles in particular have been excellent candidates for novel drug delivery vehicles because they solubilize agents either by covalently attaching them to block copolymers prior to the formation of a micellar structure or by physically entrapping the hydrophobic compound/drug within the micelle (Kazunori et al., 1993). Moreover, reduced off-target toxicity and sustainable drug release profiles can be achieved by polymeric micelle nanocarriers (Jones and Leroux, 1999; Kataoka et al., 2001; Shin et al., 2016). Cylindrical micelles lend themselves to flow intense drug delivery applications (e.g., phage-mimetic drug carriers and micropore delivery) since they are not only bio-inert and stable, but also very flexible. In general, size and shape of polymer micelles differ according to monomer selection, length of block copolymers, chain architecture, and temperature (Pochan et al., 2004; Zhulina et al., 2005). To date, a wide range of amphiphilic block copolymers have been employed in order to create copolymer micelles. Chitosan (e.g., chitosan graft poly(ε-caprolactone (PCL), N-octly-N, O-carboxymethyl chitosan), polyacrylate [e.g., poly(2-ethylhexyl acrylate)-*b*-poly(acrylic acid)], polycapro-lactone [e.g., polyethylene oxide (PEO)-b-PCL], polylactide [e.g., poly(D,L-lactide)-*b*-poly(ethylene oxide) (KríŽ et al., 1999; Lim Soo et al., 2002; Štěpánek et al., 2009; Duan et al., 2010; Huo et al., 2011) and many more examples are listed in the review by Kulthe et al. (2012). Most of these block copolymers assemble into spherical micelles whereas only a small portion of block copolymers are able to form cylindrical micelles. For example, cylindrical micelles have been produced in appropriate organic solution by self-assembly of block copolymers having either crystallizable or amorphous blocks (Nazemi et al., 2016). Polyferrocenylsilane (PFS) based block copolymers [e.g., PFS- polyisoprene (PFS-PI), PFS-polydimethyl-siloxane (PFS-PDMS) and PFS-polymethylvinylsiloxane (PFS-PMVS)]] with PFS as a crystallizable block, favor formation of well-defined, monodisperse cylindrical micelles with controllable length over a wide range of block ratios (Zhang and Eisenberg, 1995; Won et al., 1999; Cui et al., 2007; Wang et al., 2007). On the other hand, formation of cylindrical micelles by amorphous blocks is restricted to a small fraction of block copolymer compositions. In addition, resulting micelles are polydisperse and confounded by the coexistence of other types of assemblies (Nazemi et al., 2016). However, the formation of cylindrical micelles is challenging and thus, the experimental data on cylindrical micelles are limited. Therefore, in this review, we mainly focus on spherical micelles.

Micelles are produced via different methods; direct dissolution and solvent switch (Riess, 2003; Letchford and Burt, 2007). When the block copolymers are relatively water-soluble, micelles are prepared by direct dissolution of the copolymer in water: the block copolymers are simply added to aqueous solutions at a concentration above the CMC, which induces the self-assembly of copolymers into micelles (Kabanov and Alakhov, 2002; Karayianni and Pispas, 2016). When the block copolymer is not water soluble, it is dissolved in a water miscible, volatile solvent, followed by removal of the organic solvent by dialysis against water (Kim et al., 1998).

Polymer assemblies (micelles or polymersomes) can be engineered to respond to various internal stimuli (pH, redox potential, and enzymes) or external stimuli (light, magnetic field, and ultrasound), leading to the formation of responsive or “smart” polymer assemblies. Depending on the applied stimulus, observed responses (e.g., disruption of polymer assemblies, changes in shape, volume, permeation rate, and conformation) vary. One of the most investigated stimulus is pH, since changes in pH in cells and tissues are associated with specific physiological and pathological conditions. For instance, the pH in tumors (pH 5.7 to 7.2) is usually reduced compared to normal tissues (pH 7.4) (Tannock and Rotin, 1989). The most prominent pH sensitive polymer micelles are composed of poly (acrylic acid) (PAA) and its derivative poly (methacrylate acid) (PMAA) (James et al., 2014). These two polymers are hydrophilic at pH 7.4 and they become hydrophobic under acidic conditions. This feature can be exploited in the design of drug delivery vehicles. Other widely investigated and applied smart micelles are thermo-responsive polymer micelles (Torchilin, 2009). Principally, thermo-responsive micelles are obtained from thermo-sensitive polymers such as poly(N-isopropyl acrylamide) (PNIPAM) (Dimitrov et al., 2007; Lang et al., 2018). This specific polymer exhibits a lower critical solution temperature (LCST) of around 32°C in aqueous based solutions (Gorelov et al., 1997). Below its LCST, PNIPAM in water exists in an expanded coil like conformation, which gives a transparent homogenous solution. At T>LCST, PNIPAM undergoes a hydrophobic collapse marked by a conformational change to a globule state which leads to the cloudiness of the solution (Wu and Wang, 1998; Zhang and Wu, 2001). Over the last decade, new families of thermo-sensitive polymers with LCST or upper critical solution temperature (UCST) different to PNIPAM have emerged (Lutz et al., 2006; Hoogenboom et al., 2008; Glatzel et al., 2010). Examples include that are based on polyethylene glycol (PEG) and oligo(ethylene glycol) methacrylate (OEGMA) and 2-(2^−^methoxyethoxy)ethyl methacrylate (MEO_2_MA) (Lutz and Hoth, 2006; Lutz, 2011). In particular, poly(MEO_2_MA), PMEO_2_MA, shows an LCST of around 26°C in water, which limits applications *in vivo*, whereas the LCST of poly(OEGMA), POEGMA, is around 90°C (Han et al., 2003; Mertoglu et al., 2005). Therefore, p(MEO_2_MA-OEGMA) is expected to have an LCST above 26°C and below 90°C. Indeed, an LCST of 37°-39°C has been obtained with 8 or 10 % of OEGMA in the initial polymer mixture (Lutz and Hoth, 2006). P(MEO_2_MA-OEGMA) copolymer exhibits an expanded coil state when it is hydrated well below the LCST. With increasing temperature, the co-polymer undergoes a coil to globe transition (Santos et al., 2018). Further approaches for designing responsive polymer micelles have been reviewed elsewhere (Schmaljohann, 2006; Stuart et al., 2010; Felber et al., 2012; Li et al., 2013; Jhaveri and Torchilin, 2014; Bordat et al., 2018).

### Polymer Micelles Equipped With Biomolecules

In order to create bio-functional polymer micelles, a variety of biomolecules including proteins, peptides, nucleic acids, and phospholipids can be chemically conjugated to or physically entrapped in the micelles. Principally, depending on their nature, biomolecules can be either conjugated to the hydrophilic part (also known as shell or corona) or incorporated into the hydrophobic part (core) of the micelles. Functionalization of the shell by biomolecules modifies the overall physicochemical and biological properties of the micelles, leading to the design of novel nanocarriers for targeted drug delivery applications (Blanazs et al., 2009).

To harness polymeric micelles with biomolecules two basic approaches apply. The first involves the physical entrapment of hydrophobic biomolecules or drugs like DOX in the core (Kataoka et al., 2012). This occurs by adding the molecule of interest during the micelle formation. The addition of the hydrophobic biomolecule does not interfere with the integrity and the stability of the micelles (Cabral and Kataoka, 2014). The alternative is the direct chemical conjugation or surface decoration of the micelle with a biomolecule provided the surface-exposed polymer block and the biomolecule bear functional groups that allow a chemical reaction. There are many combinations of polymer micelle-biomolecule conjugates. For instance, polymer-based micelles containing deprotected aldehyde groups were able to chemically bind RGD peptides (Duong et al., 2010). In this case the chemical conjugation of micelles with peptides not only does not affect negatively their self-assembly, but also enhances their biocompatibility and eases their uptake from mammalian cells (Ukawala et al., 2011; Han et al., 2017). Furthermore, this strategy applies also to the use of polymer micelles for siRNA delivery and micelle-protein conjugates (Amjad et al., 2017; Han et al., 2017).

To date, many distinct biomolecules have been conjugated to block copolymer micelles. The most commonly used biomolecules include proteins (Torchilin, 2004; Holliger and Hudson, 2005; Zeng et al., 2006; Skidan et al., 2009; Sawant et al., 2013; Fan et al., 2017), peptides (Kamaly et al., 2012), and sugar moieties (Yasugi et al., 1999; Nagasaki et al., 2001; Jule et al., 2003; Oishi et al., 2006). In some cases, they function as targeting ligands that specifically recognize antigens or receptors that are overexpressed on cancer cells, leading to active targeting in cancer therapy. For example, antibodies to cell-specific surface molecules are conjugated to polymer micelles to mediate a specific localization of the carrier (Torchilin, 2004; Holliger and Hudson, 2005). More specifically, the doxorubicin-loaded polyethylene glycol-phosphatidyl ethanolamine (PEG-PE) micelles were decorated with a monoclonal 2C5 antibody, and were shown to recognize several types of tumor cells (Perche et al., 2012). Targeting of these “immunomicelles” (2C5-MDOX) was evaluated using an ovarian cancer cell spheroid model. The superior accumulation of 2C5-MDOX compared to free doxorubicin or untargeted MDOX in spheroids revealed itself by a more efficient penetration of the tumor and an increase in cytotoxicity. In another example, two anti-cancer drugs, paclitaxel and campthothecin, were specifically loaded to 2C5 conjugated, mixed PEG-PE/vitamin E micelles and their cytotoxicity was tested in cancer cells *in vitro* (Sawant et al., 2008). The vitamin E increased drug loading efficiency due to its ability to solubilize hydrophobic molecules within the mixed micelles. Correspondingly, drug loaded mixed immunomicelles were more cytotoxic. In another example, anti-Her2 antibody Fab fragment conjugated to temperature-responsive, poly(N-isopropylacrylmide-*co*-N,N'-dimethylacryl-amide)_118_-*b*-poly(_D,L_-lactide)_71_ (PID_118_-PLA_71_) micelles lead to the formation of immunomicelles with dual targeting function (Li et al., 2012). Experimental data showed that the cooperative effects of both temperature and Fab moiety significantly enhanced the cytotoxicity *in vitro*. These immunomicelles also showed elevated stability and intratumor accumulation in tumor bearing mice and finally, significant *in vivo* tumor inhibition.

The transferrin receptor is a very attractive target protein since it is over-expressed in many cancer cells (Singh, 1999). Accordingly, polymer micelles have been modified with either transferrin, the endogenous ligand, or antibodies against the transferrin receptor. Compared to non-conjugated micelles and free R547, transferrin conjugated PEG2000-PE micelles loaded with R547 showed increased *in vitro* interaction with ovarian carcinoma cells that highly express transferrin receptors (Sawant et al., 2013). Transferrin-conjugated micelles also showed enhanced cytotoxicity *in vitro* and an appreciable inhibition of tumor growth compared to drug loaded micelles lacking transferrin on their surface. Furthermore, polymer micelles have been armed with tumor necrosis factor related apoptosis inducing ligand (TRAIL) (Skidan et al., 2009) and epidermal growth factor (EGF) (Zeng et al., 2006) as targeting moiety.

Peptides are smaller targeting ligands with lower immunogenicity and better *in vivo* stability (Kamaly et al., 2012). For example, cyclic arginine-glycine-aspartic acid (cRGD) conjugated polymer micelles (PMs) made from PEG-*b*-poly (_L_-glutamic acid) and (1, 2-diaminocyclohexane) platinum (II) (DACHPt) have been used for the delivery of anti-cancer drugs to gliobastoma (Miura et al., 2013). Compared with the corresponding PMs bearing non-targeted, “cyclic-RAD” (cRAD) ligand, cRGD-PMs achieved more efficient drug delivery to tumors in the mouse model of U87MG human glioblastoma. Intravital confocal laser scanning microscopy revealed that the cRGD-linked PMs had high permeability from vessels into the tumor parenchyma where they rapidly accumulated. In another example, RGD peptide conjugated to the surface of poly(ethylene oxide)-*b*-poly(ε-caprolactone) (PEO-*b*-PCL) micelles mediated targeting of the drug delivery by binding to the integrins overexpressed on the surface of metastatic cancer cells (Xiong et al., 2007). Other peptides used to modify PMs include Lyp-1 (Cys-Gly-Asn-Lys-Arg-Thr-Arg-Gly-Cys) (Wang Z. et al., 2012) cell penetrating peptides, the transcriptional transactivator (TAT) from HIV-1 (Kanazawa et al., 2012), and the somatostatin mimic octreotide (Xu et al., 2013). Sugars, e.g., glucose and galactose (Yasugi et al., 1999), linked to PMs have also been reported to target drug delivery, in particular to immune cells. In a later study, 1-O-substituted lactose and mannose were also successfully conjugated to poly(ethylene glycol)–poly(D,L-lactide) (PEG-PLA) micelles (Nagasaki et al., 2001). Both, galactose and lactose mediated the interaction of PEG-PLA micelles with *Ricinus communis* agglutinin 1 (RCA-1) lectin, whereas binding to concanavalin A (Con A) lectin was mediated only by the specific ligand mannose.

The growing trend is to design multifunctional polymer micelles that combine several of the biomolecules described above with stimulus-responsive block copolymers into a single nanocarrier. It is expected that such multifunctional micelles can improve the efficacy of current carriers, not only for delivering small molecules, but also for other biologics including therapeutic genes and antibodies.

## Polymersomes

Polymersomes are membrane-enclosed 3D supramolecular structures formed by self-assembly of corresponding amphiphilic block copolymers in aqueous solutions (Discher et al., 1999). They have a similar membrane morphology to liposomes (phospholipid vesicles). However, they have several advantages over liposomes, partially owing to the larger molecular weight of the block copolymer. Notably, the synthetic nature of amphiphilic block copolymers enables controlling the properties of the polymersomes produced (Discher and Eisenberg, 2002), such as membrane thickness and fluidity (Discher and Ahmed, 2006; Itel et al., 2014), permeability (Battaglia et al., 2006; Rodríguez-García et al., 2011), and stability (Rodríguez-García et al., 2011). For example, the membrane thickness of liposomes is typically 3–5 nm whereas polymersome membranes can be tuned in the range of ~8–21 nm (Bermudez et al., 2002). More specifically, the membrane thickness of polymersome depends on the molecular weight of polymers and degree of polymerization, principally hydrophobic blocks (Winzen et al., 2013). In terms of diameter, liposomes and polymersomes can both form nanometer sized vesicles or giant vesicles (>1 μm) depending on the methods applied for vesicle preparations (Kita-Tokarczyk et al., 2005). Polymersomes are predominantly prepared by: (i) film rehydration, (ii) solvent switch, and (iii) direct dissolution. In the film rehydration method, the block copolymers are first dissolved in appropriate organic solvent which is then evaporated either with a stream of nitrogen or by applying a vacuum in a rotary evaporator. The resulting thin copolymer film is rehydrated by addition of an aqueous solution under continuous stirring. Solvent switch and direct dissolution methods correspond to those described above for the preparation of micelles. These commonly used methods as well as electroformation, double emulsion and microfluidics methods have also been applied to prepare GUVs from block copolymers (Discher et al., 1999; Thiele et al., 2010), but these micron sized vesicles are not discussed in this review.

Based on membrane thickness and vesicle size, the internal volumes of both lipid and polymer vesicles have been compared (Rideau et al., 2018). For small and large lipid vesicles, the internal volume is small (>10–9 μL) whereas for polymersomes the variations in internal volume is high due to difference in membrane thickness. However, membrane thickness is less affective for internal volume of the small and large lipid vesicles compared to vesicle size. For giant lipid and polymer vesicles, the effect of membrane thickness on internal volume is negligible. negligible. In contrast to liposomes whose lipids are subject to oxidation, polymersomes are more stable and can have a significantly longer shelf live (Discher et al., 2007). The intrinsic membrane properties of polymersomes and liposomes have been extensively studied and compared (Le Meins et al., 2011). Moreover, there are special, recently developed polymersomes based on a polyion complex, named PICsomes (Koide et al., 2006) and capsosomes (Städler et al., 2009). PICsomes are formed by simply mixing oppositely charged block copolymers in an aqueous solution (Koide et al., 2006). They are prepared by mixing for example anionic PEG-poly(α,β-aspartic acid) and cationic PEG-poly([2-aminoethyl]-α,β-aspartamide) in aqueous solution (Anraku et al., 2010). PICsomes have also been loaded with enzymes (e.g., l-asparaginase or βgalactosidase) for enzyme delivery (Anraku et al., 2016; Sueyoshi et al., 2017).

Capsosomes are polymer carrier capsules containing liposomal subcompartments. They are formed by the layer-by-layer deposition of polymers and liposomes on sacrificial template particles (e.g., silica) followed by removal of the template particles (Maina et al., 2015). Specifically, a polymer precursor layer [e.g., poly(L-lysine) (PLL) or poly (methacrylic acid)-co-(cholesteryl methacrylate) (PMA_c_) or mixture of both] and liposomes [e.g., zwitterionic 1,2,-dioleoyl-sn-glycero-3-phosphocholine (DOPC) liposomes] are deposited on the template, followed by the alternating deposition of separation layers and liposomes until the desired number of layers is reached (Chandrawati et al., 2010; Hosta-Rigau et al., 2014). Capsosomes have been functionalized either by incorporating a small hydrophobic model peptide into the membrane of subcompartments or by encapsulating enzymes into subcompartments (Hosta-Rigau et al., 2014). However, details of PICsomes and capsosomes and their biomolecule conjugation will not be addressed in this review.

To date, many different amphiphilic block copolymers have been synthetized that form polymersomes. Some of the most commonly used block copolymers are poly(ethyl ethylene)-b-poly(ethylene oxide) (PEE-*b*-PEO) (Bermudez et al., 2004), poly(butadiene)-*b*-poly(ethylene oxide) (PBD-*b*-PEO) (Discher and Eisenberg, 2002), poly(styrene)-*b*-poly(acrylic acid) (PS-PAA) (Burke et al., 2001; Discher and Eisenberg, 2002), poly(2-methyloxazoline)-*b*-poly(dimethylsiloxane)-*b*-poly(2-methyloxazoline) (PMOXA-*b*-PDMS-*b*-PMOXA) (Taubert et al., 2004), commercially available poly(propylene oxide)-based (PPO) architectures (PEO-*b*-PPO-*b*-PEO, pluronics) (Rodríguez-García et al., 2011), and poly(ethylene oxide)-*b*-polycaprolactone-*b*-poly(2-methyl-2-oxazoline) (PEO-*b*-PCL-*b*-PMOXA) (Konishcheva et al., 2017).

### Polymersomes Equipped With Biomolecules

A variety of biomolecules have been combined with polymersomes either by conjugating them to the outer surface of polymersomes, by inserting them into the polymeric membrane or by encapsulating them inside the polymersome cavity. A large diversity of chemical functionalization can be realized by the control of block copolymer synthesis. Conjugating biomolecules to the outer surface of polymersomes can be achieved by covalent or non-covalent attachment using conventional immobilization techniques. For covalent attachment of biomolecules, reactive or functional end groups such as hydroxyl, amine and N-hydroxlsuccimidyl esters are first introduced to the end of the hydrophilic domain of the block copolymer during its synthesis. To form polymersomes, the end-functionalized block copolymer is mixed with a non-functionalized one since the end-functionalization changes the packing parameter of the block copolymer and thus, their self-assembly characteristics in aqueous solution.

**Figure 3:**
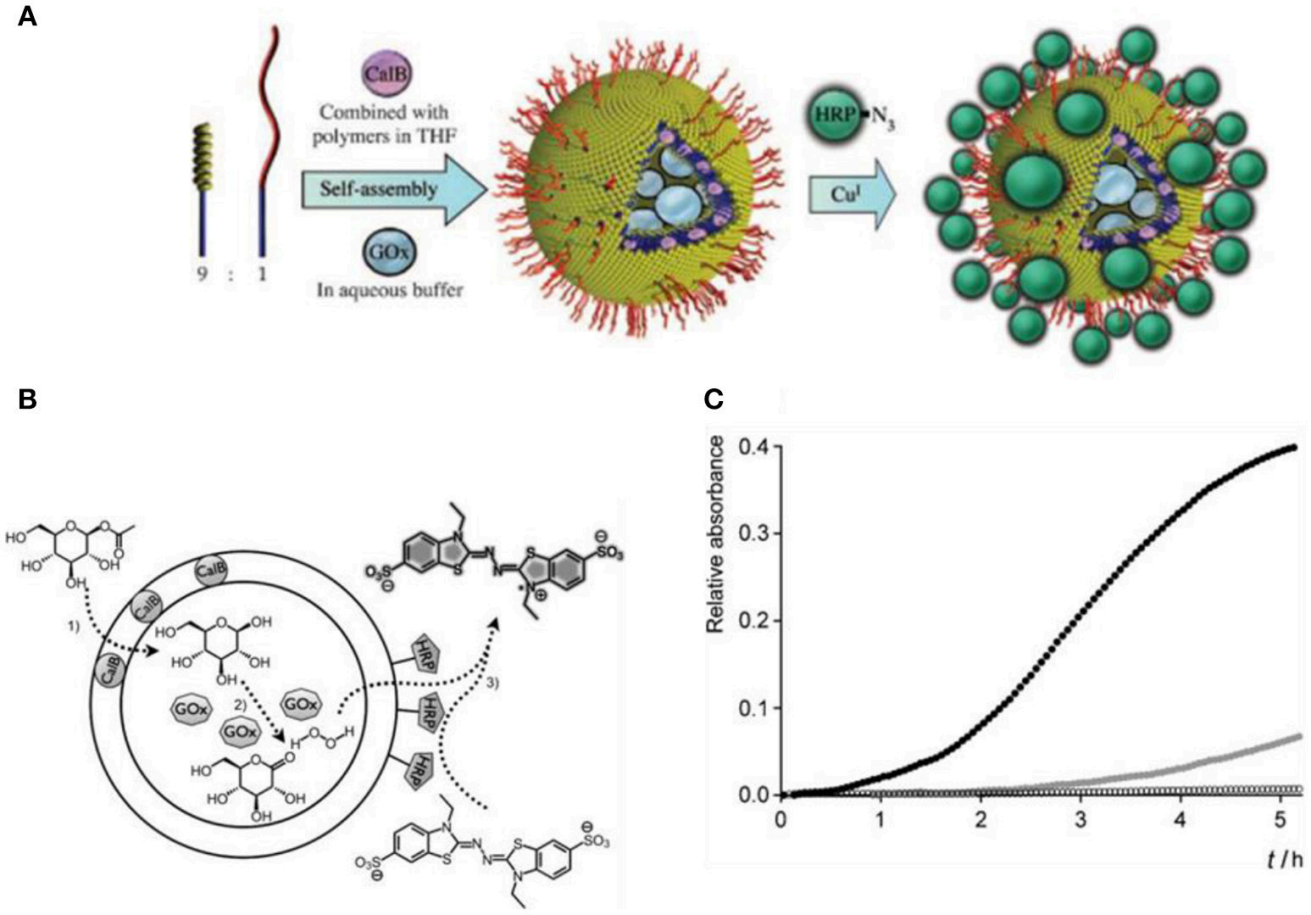
Positional assembly of enzymes in a polymersome. **(A)** A mixture of PS-PIAT and anchor is lyophilized with CalB and then dissolved in THF. This mixture is then injected into an aqueous buffer containing GOx, encapsulating it in the inner compartment and subsequently trapping CalB in the polymeric bilayer. A third enzyme, HRP, is immobilized on the polymersomal perimeter though a covalent linkage to an anchor, creating an outer shell of enzymes. **(B)** Schematic representation of the multi-step reaction, and **(C)** Progress curve for the three enzyme cascade reaction. Reproduced from van Dongen et al. (2009) with permission of Copyright © 2009 WILEY-VCH Verlag GmbH & Co. KGaA, Weinheim.

So far, the covalent attachment of biomolecules to polymersomes has been achieved by various methods, such as by click chemistry based on azide–alkyne cycloaddition (van Dongen et al., 2009; Deng et al., 2016; Anajafi et al., 2017), vinyl sulfonyl coupling with amines (Petersen et al., 2010), coupling of *N*-hydroxysuccinimidyl esters with primary amines (Christian et al., 2007; Egli et al., 2011), and maleimide with thiol groups (Pang et al., 2008; Lu et al., 2017). Inspired by nature, polymer based catalytic nanocompartments (CNCs) can be designed to carry out chemical, enzymatic, and even cascade reactions. Polymersomes are especially suited to constitute a nanocompartment since the bounding polymer membrane protects the encapsulated enzymes and helps to maintain their activity. For example, horseradish peroxidase (HRP) was linked to the surface of polymersomes through azide–alkyne cycloaddition click chemistry by using a polystyrene-*b*-poly (l-isocyanoalanine (2-thio-phen-3-yl-ethyl) amide) (PS-*b*-PIAT) block copolymer and a block copolymer with an acetylene-functionalized hydrophilic terminus (Figure 3; van Dongen et al., 2009). Another two enzymes were combined with different parts of the HRP-polymersomes, *Candida antarctica* lipase B (CalB) was incorporated into the membrane, and glucose oxidase (GOx) was encapsulated inside the cavity. These enzymes sequentially converted glucose acetate to glucose which was further oxidized to gluconolactone. The hydrogen peroxide produced in this reaction was detoxified by HRP mediated oxidation of 2,2′-azinobis (3-ethylbenzothiazoline-6-sulfonic acid (ABTS) to ABTS^+^(van Dongen et al., 2009).

In another example, vinyl sulfone functionalized poly(ethylene oxide)-*b*-poly (g-methyl-3-caprolactone), VS-PEO-*b*-PMCL, block copolymers self-assembled into polymersomes to which thiol-containing CGRGS targeting peptides were conjugated under mild conditions in the absence of a catalyst (Petersen et al., 2010). Peptide coupling did not change the morphology of the polymersomes. Furthermore, 6-hydrazinocotinate acetone hydrazone (HyNic) modified Trastuzumab antibodies were conjugated to the surface of 4-foramybenzoate (4FB) functionalized PDMS-b-PMOXA polymersomes by coupling of *N*-hydroxysuccinimidyl esters with primary amines (Egli et al., 2011). In a similar concept, thiolated mouse anti-rat monoclonal antibody (OX26) was covalently attached to the surface of maleimide-functionalized poly(ethylene glycol)-*b*-poly (ε-caprolactone) (PEG-*b*-PCL) polymersomes (Pang et al., 2008).

Strategies for the non-covalent attachment of biomolecules on polymersome surfaces include biotin-avidin interactions (Lin et al., 2004, 2006; BroŽ et al., 2005; Hammer et al., 2008), metal/nitrilotriacetic acid (NTA)–histidine interaction (Nehring et al., 2008, 2010; Tanner et al., 2012), and host-guest inclusion complexation between β-cyclodextrin and adamantane (Felici et al., 2008; Guo et al., 2008). For example, the terminal end of OB29 block copolymer was functionalized with 4-fluoro-3-nitrobenzoic acid (4F3 NB) followed by the addition of biotin (Hammer et al., 2008). By functionalizing the outer shell of the polymersome with biotin, the modular avidin–biotin chemistry has been used to bind NeutrAvidin, which served to attach the biotinylated cell adhesion molecules, selectin and integrin. Alternatively, NeutrAvidin-coated polymersomes served to bind biotinylated-sLe^x^, biotinylated-anti-ICAM-1(b-anti-ICAM-1), or a mixture of the two. Binding studies on surfaces bearing only ICAM-1/Fc, ICAM-1/Fc and P-selectin/Fc, or P-selectin/Fc alone revealed that vesicles bearing both ligands showed superior adhesion. Another approach of surface attachment was reported for PB-*b*-PEO block copolymer-based polymersomes with terminal lysine-NTA functional groups which were treated with metal ions such as Cu^2+^ and Ni^2+^ to attract polyhistidine-tagged proteins before their self-assembly into polymersomes (Nehring et al., 2008). Then, polyhistidine-tagged proteins such as fluorescently labeled maltose binding protein (His_10_-MBP-FITC), polyhistidine-tagged enhanced green fluorescent protein (His_6_-EGFP) and polyhistidine-tagged red fluorescent protein (His_6_-RFP) were coupled to metal-NTA functionalized polymersomes. Similarly, fluorescently labeled histidine-tagged peptides were selectively attached to the surface of Cu^2+^-Tris-NTA containing PB-*b*-PEO polymersomes to mediate targeting (Tanner et al., 2012).

In the case of host-guest inclusion complexation, host groups [e.g., β-cyclodextrin (β-CD)] and guest groups are included at both ends of block copolymers that self-assemble into polymersomes. More specifically, the building blocks of polyether imide (PI) with β-CD at both ends (CD-PI-CD) self-assembled into polymersomes. The presence of β-CD at the surface of polymersomes enabled the formation of inclusion complexes with adamantane-ended polyethylene glycol (PEG) (Guo et al., 2008). In another example, polystyrene (PS) appended with β-CD formed polymersomes to which HRP molecules that were modified with adamantane groups through a PEG spacer were conjugated (Felici et al., 2008). Enzyme activity assays showed that HRP preserved its catalytic activity after the attachment to the polymersomes.

**Figure 4:**
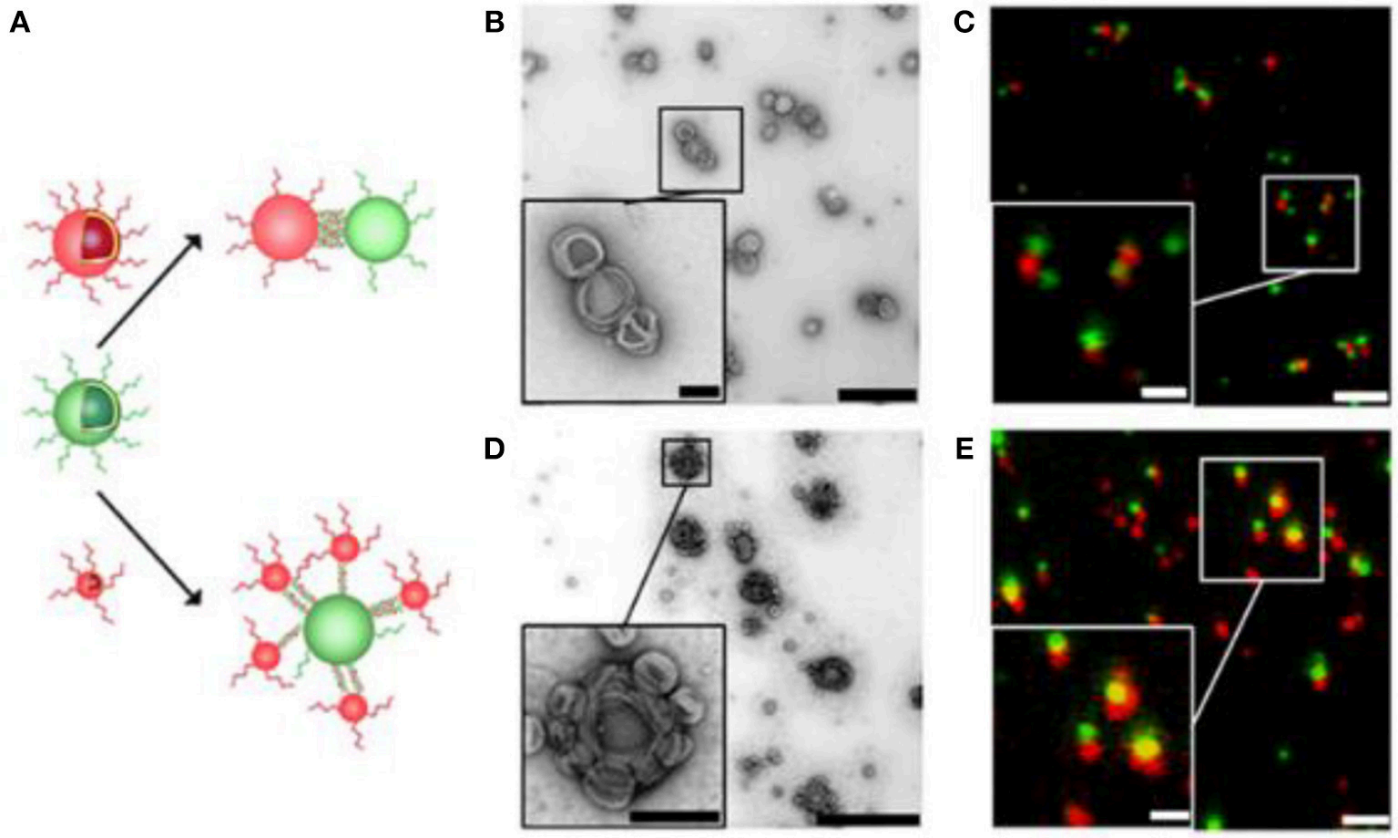
Self-organization of complementary ssDNA-polymersomes. **(A)** Schematic representation of distinct spatial topology resulting from mixing differently sized complementary ssDNA-polymersomes. TEM and CLSM micrographs of chain-like **(B,C)** and satellite-like polymersome clusters **(D,E)**. The scale bar for TEM micrographs is 1,000 and 200 nm in the inset; in CLSM micrographs it is 2 and 1 μm in the inset. Reproduced from Liu et al. (2016) with permission from Copyright (2016) American Chemical Society.

Besides conjugating biomolecules to preformed functionalized polymersomes, block copolymers were co-synthesized together with biomolecules such as the green-fluorescent protein variant amilFP497 (Wong et al., 2015) and heparin (Najer et al., 2014). More specifically, amilFP497 was linked to thermo-responsive poly(N isopropyl acrylamide) (PNIPAM) and heparin was linked to PDMS. The protein-polymer bioconjugates were then mixed with block copolymers to form biomolecule-decorated polymersomes. With this approach, an enhanced coupling efficiency was achieved. Other block copolymers were grafted to nucleotide sequences and the resulting DNA-block copolymers formed polymersomes (Cottenye et al., 2012; Kedracki et al., 2014). Alternatively, DNA was attached to the surface of polymersomes based on a mixture of PMOXA-PDMS-PMOXA and azide functionalized PMOXA-PDMS-PEG-N_3_ (Liu et al., 2016). The functional azide groups enabled coupling of dibenzocyclooctyne (DBCO)-derivatized single stranded DNA or its complementary oligonucleotide to the surface of polymersomes. Hybridization of complementary DNA strands was exploited as driving force to self-organize polymersomes. Specifically, polymersomes were connected via single stranded DNA molecules of 12nm length creating “polymersome clusters” (Figure 4). Conceivably, these polymersome clusters may serve as a general platform for applications such as enzyme cascade reactions.

**Figure 5:**
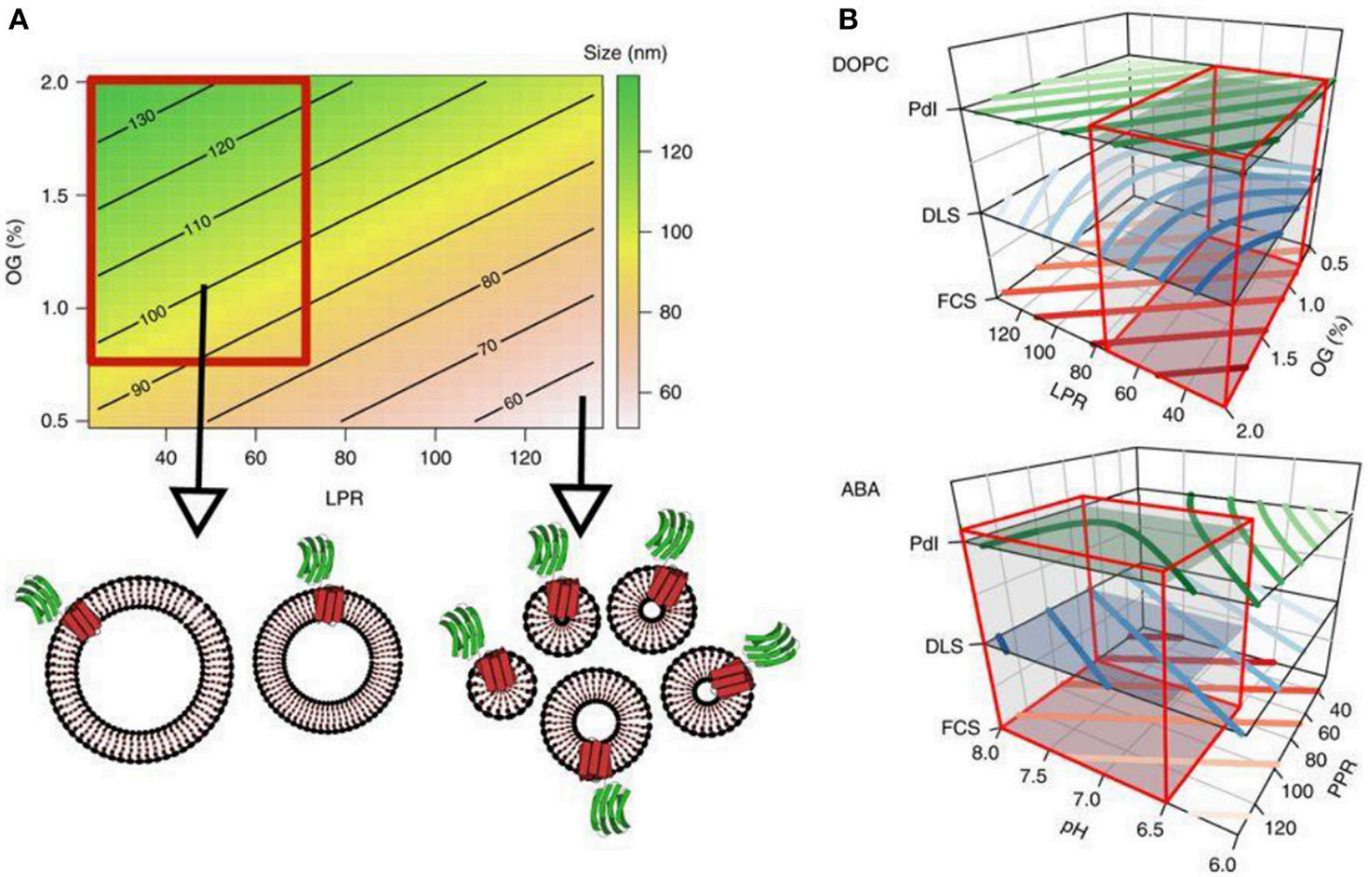
Contour plots. **(A)** The contour plot visualizes the proteoliposome size, modeled from the first round of experiments depending on the LPR and OG concentration, whereas the pH value is fixed at 7. The color gradient indicates different sizes and shows a trend toward a region within the design space which yields homogeneous, large proteoliposomes (highlighted by the red box). **(B)** This region of interest applies to all three responses as shown in the stacked contour plots for both membrane types. Red boxes encompass the subregions of the design space which was subsequently screened for proteovesicles with functioning PR-GFP. Reproduced from Goers et al. (2018) with permission of https://creativecommons.org/licenses/by/4.0/.

If the biomolecule is a membrane protein, it is normally associated with the hydrophobic part of the polymersome, i.e., tends to insert into the membrane. In case the biomolecule is hydrophilic, it will either locate to the aqueous cavity of the polymersome (i.e., be encapsulated) or to the inner and/or outer surface of the polymersome membrane. When inserting membrane proteins into synthetic membranes, it is critical to preserve their functionality. Recently, the functional reconstitution of proteorhodopsin (PR) was achieved (Goers et al., 2018). The detergent n-octyl-β-D-glucopyranoside (OG) was used to destabilize the membrane of preformed polymersomes and thereby enable the entry of PR while preserving its intrinsic functionality. To optimize this system, a comprehensive analysis of parameters underlying the reconstitution process was carried out. This study provides the basis for exploring a general mechanism of membrane protein reconstitution into synthetic membranes (Figure 5).

Impermeable polymersome membranes can be rendered permeable by the insertion of natural ion channels such as alpha-hemolysin (αHL), the carboxylic acid ionophore ionomycin (Lomora et al., 2015), or gramicidin A (gA). Permeabilization enables transport of ions and small molecules across the polymersome membrane, e.g., enzyme substrate and product diffusion to and from the polymersome cavity. For example, phosphoglucomutase (PGM), which catalyzes the conversion of glucose-1-phosphate (G1P) to glucose-6-phosphate (G6P), that was encapsulated in PMOXA-PDMS-PMOXA polymersomes was able to act inside because the membrane was permealized with aHL (Lomora et al., 2017). Alternatively, to allow the passage of larger substrates and products, bacterial outer membrane proteins (Omp) and more specifically, the porin OmpF, which forms pores for molecules with a molecular mass up to ~600 Da (Koebnik et al., 2000), have been inserted in the membrane of polymersomes (Einfalt et al., 2015). To achieve membrane insertion, OmpF can be combined with the block copolymer before film formation or it can be added to the rehydration solution before polymersome self-assembly. Pore functionality was demonstrated by loading the hydrophilic cavity with horseradish peroxidase (HRP) and testing for enzymatic activity on externally added substrates. Likewise, the molecular flow through OmpF pores rendered pH-sensitive was triggered in response to a pH change in the local environment and a reversible on/off switching of HRP activity in the cavity of the polymersomes was observed (Edlinger et al., 2017). More recently, fungal uricase and HRP were combined to function in sequence by encapsulating each of the two enzymes in separate nanocompartments that were both equipped with OmpF as a gateway for substrates and products (Belluati et al., 2018). When these catalytic nanocompartments (CNCs) were added to cells exposed to uric acid, detoxification of uric acid occurred by fungal uricase through the presence of hydrogen peroxide (H_2_O_2_). Combining distinct enzymes in separate nanocompartments opens new doors to mimic complex enzymatic pathways in a controlled fashion with a high potential in diagnostics and therapeutics.

### Polymersomes Immobilized on a Surface

Solid supported polymer-based nanoscale structures can be described as block copolymer assemblies, e.g., polymersomes or micelles that are immobilized via specific methods on a solid support. The resulting surfaces hold a tremendous potential for applications in fundamental material sciences and, when equipped with the corresponding biomolecules, as a new type of interface with which enhanced catalysis can be achieved. We will first discuss from a fundamental physical chemistry point of view how immobilization of polymersomes on solid supports such as glass, mica and silica can be achieved. We then move to more advanced systems where polymersomes are not only immobilized on a surface, but they are also able to carry out distinct functions and respond to external stimuli due to their conjugation with corresponding biomolecules.

Immobilization of polymersomes on a solid support not only creates a more robust system but also offers the possibility to control topology. Toward this goal, click chemistry has been predominantly employed to immobilize polymersomes on solid supports. Click chemistry refers to organic reactions that allow the connection of two synthetic molecular building blocks or to bioconjugation if at least one of the partners is a biomolecule. Click chemistry involves some of the most common reactions of modern organic and polymer chemistry, such as Diels–Alder, Michael addition, pyridylsulfide reaction, oxyme, thiolene, strain-promoted azide–alkyne cycloaaddition (SPAAC), and Cu(I)-catalyzed azide–alkyne cycloaddition (CuAAC) (Castro et al., 2015).

**Figure 6:**
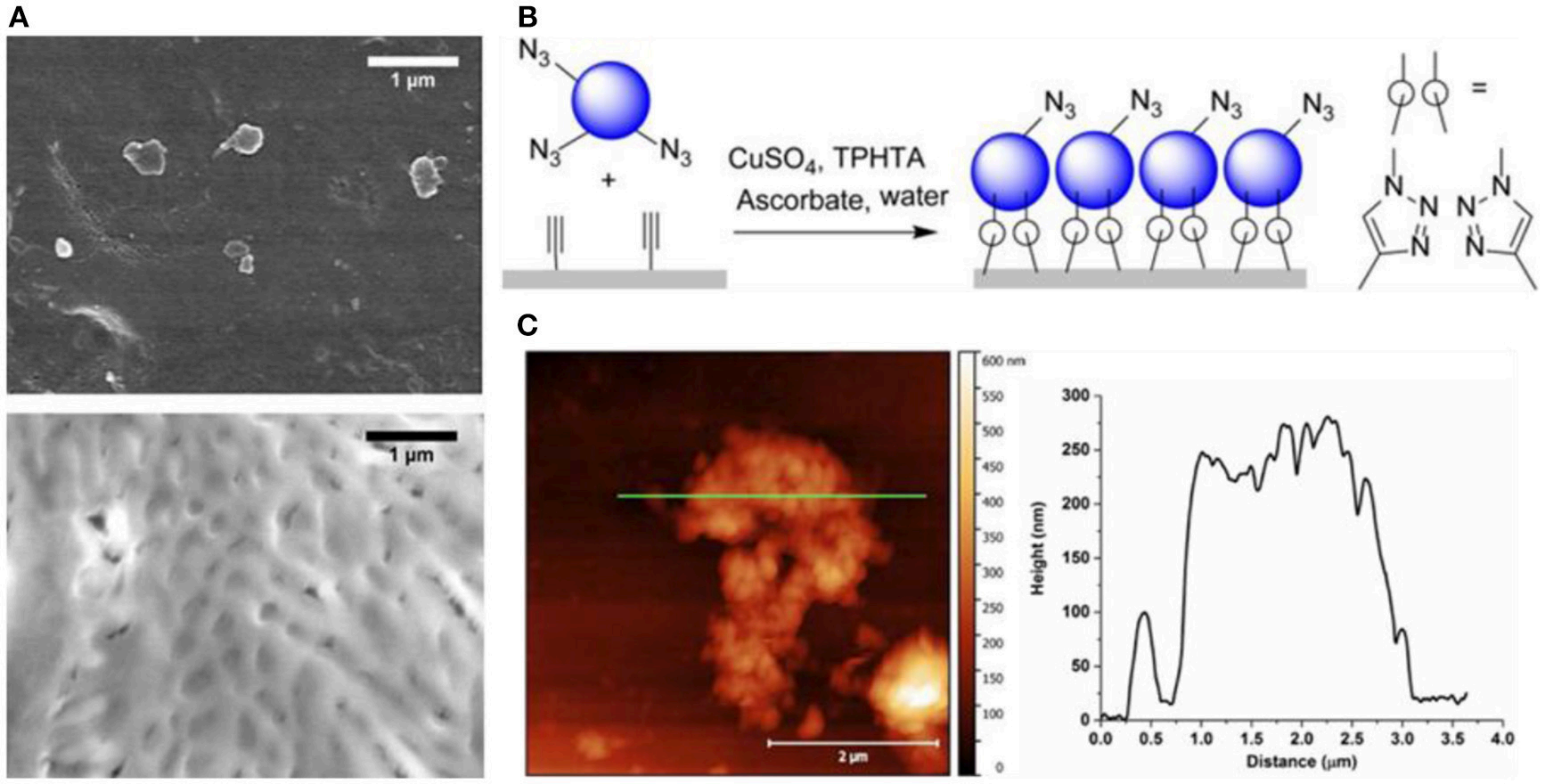
Polymersome attachment on the alkyne modified PAN membrane surface. **(A)** Cryo-SEM images of membrane supported immobilized polymersomes after extensive washing. *Top*, Amine-terminated ABA polymersomes on hydrolyzed PAN membranes. The membrane pores are visible. *Bottom*, Azide-terminated ABA polymersomes on alkyne-functionalized PAN. A continuous layer of collapsed polymersomes can be observed. **(B)** Schematic representation of polymersome-clicking on the alkyne modified PAN membrane surface. **(C)** *left*, AFM of a PAN membrane covered with polymersomes immobilized by CuAAC on alkyne-functionalized PAN. A partially covered area was selected to determine the layer thickness and the shape of assembled polymersomes. AFM cross section (green line) is shown on the right. Reproduced (with adaptations) from Rein et al. (2016) with permission of Copyright © 2016 Wiley Periodicals, Inc.

The use of click chemistry for the attachment of polymersomes on a solid support requires a functionalized polymer to expose specific molecular groups and a chemically treated surface. A recent example reports immobilization of polymer vesicles assembled from a triblock copolymer poly(2-methyloxazoline)-poly(dimethylsiloxane)-poly(2-methyloxazoline) (PMOXA-PDMS-PMOXA) terminated with either carboxylate- or amino groups, on click-functionalized porous polyacrylonitrile (PAN) membranes (Rein et al., 2016). The coupling of the polymersomes on the PAN surfaces involves the azide functionalization of the polymersomes followed by the surface conversion of the alkyne groups of the substrate to triazoles, which then results in a chemical bond between the solid support and the polymersomes (Figure 6). Polymersomes immobilized on solid substrates can be monitored by several microscopy techniques including atomic force microscopy (AFM), cryo-scanning electron microscopy (Cryo-SEM), and fluorescence microscopy. In addition to revealing the topography of surface-attached polymersomes in buffer with minimal perturbation, AFM allows for measuring the thickness of the vesicle-based layer. The Cryo-SEM was used to characterize the vesicle adlayer on the surface as a complementary method to AFM, but due to the harsh sample preparation conditions (platinum coating, high vacuum etc.), the morphology of the polymersomes was mostly disrupted. Nevertheless, a high coverage of the substrate with polymersomes was observed when the PAN surface and the polymersomes were modified with alkyne and azide functional groups, respectively. Consistent with the presence of available alkyne-groups, fluorescence microscopy of PAN coated substrates stained with an azido-coumarin dye revealed an increase of the fluorescence signal after modification. By encapsulating the fluorescent dye 2-(N-methyl-N-2-4-carboxyl butyl amino (6,10-bis(N,N-dimethylamino) trioxatriangulenium chloride (ATOTA-COOH) in the polymersomes, immobilization on the alkyne-modified PAN surfaces has been demonstrated. Recently, by using a combination of SPAAC and thiol-ene reactions, different polymersomes and mixtures of polymersomes and micelles have been co-immobilized on a solid support (Rigo et al., 2018). PDMS-based polymersomes served as an ink for creating locally defined patterns on glass, leading to multifunctional surfaces with controlled properties.

A general procedure for the immobilization of polymersomes on solid silica substrates via a biocompatible thiol–ene reaction has recently been developed (Gunkel-Grabole et al., 2017). This reaction takes place with copper-free catalysis to avoid the cytotoxicity of transition metals (Cortizo and De Mele, 2004). Methacrylate end group functionalized PMOXA-PDMS-PMOXA polymersomes were immobilized on thiol-functionalized surfaces with or without with polyethyleneglycol (PEG) spacers in presence of Tris(2-carboxyethyl)phosphine hydrochloride (TCEP) as a catalyst.

Another method for the immobilization of polymersomes on a solid support is by taking advantage of electrostatic interactions. For example, polymersomes have negatively charged functional groups and the substrate is positively charged or vice versa. Accordingly, polymersomes based on pluronic L121 triblock copolymers (PEO5-PPO68-PEO5) were mixed with high molecular weight poly(acrylic acid) (PAA), which is negatively charged at neutral pH, for immobilization onto a glass or mica surface (Li et al., 2008). The immobilization was achieved by Mg^2+^-mediated electrostatic interactions between the polymersomes and the solid support. Noticeably, the polymersomes “gently” attached to the surface at room temperature and detached from the surface with increasing temperature. In a similar approach, amine-functionalized polymersomes assembled from poly(lactide-*b*-ethylene oxide) (PLA-PEO), poly(capro-lactone-*b*-ethylene oxide) (PCL-PEO), and poly(isoprene-*b*-ethy-leneoxide) (PI-PEO). PLA-PEO and PCL-PEO were attached to aldehyde-functionalized glass surfaces (Domes et al., 2010). The different chemical composition of the polymers led to different physical and chemical properties of the polymersomes. The PCL-PEO polymersomes were stiffer than PI-PEO, because they remain glassy at room temperature (Tg = 60°C). These differences affected not only the shape of the polymersomes but were also reflected in the footprint area, i.e., the contact area between the polymersome and the solid support. Bound PEO-PCL polymersomes showed a small, deformed footprint area and a barrel-type shape while the high bilayer flexibility of PI-PEO resulted in a larger footprint area and a hollow spherical structure without significant deformation.

**Figure 7:**
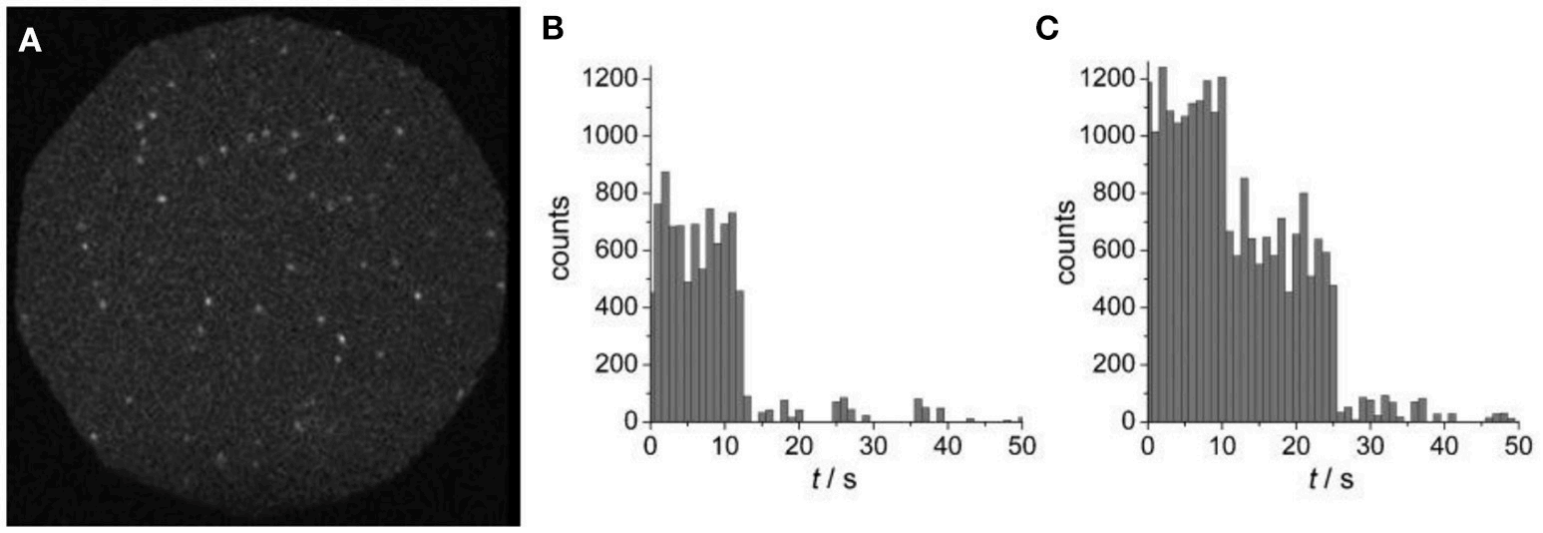
**(A)** Wide-field fluorescence image of surface-tethered polymersomes containing Atto655-labeled PGK. **(B)** Single-step, and **(C)** two-step photobleaching events are presented as time courses of fluorescence emission intensities, as obtained by integrating individual spots. Reproduced from Rosenkranz et al. (2009) with permission of Copyright © 2009 WILEY-VCH Verlag GmbH & Co. KGaA, Weinheim.

Yet another approach for immobilizing polymer based nanostructures on a solid substrate is based on streptavidin-biotin interaction (González et al., 1997). Streptavidin adsorbed to a glass substrate was shown to interact with biotinylated tri-block copolymer vesicles made from PMOXA-PDMS-PMOXA (Figure 7) (Rosenkranz et al., 2009).

**Figure 8:**
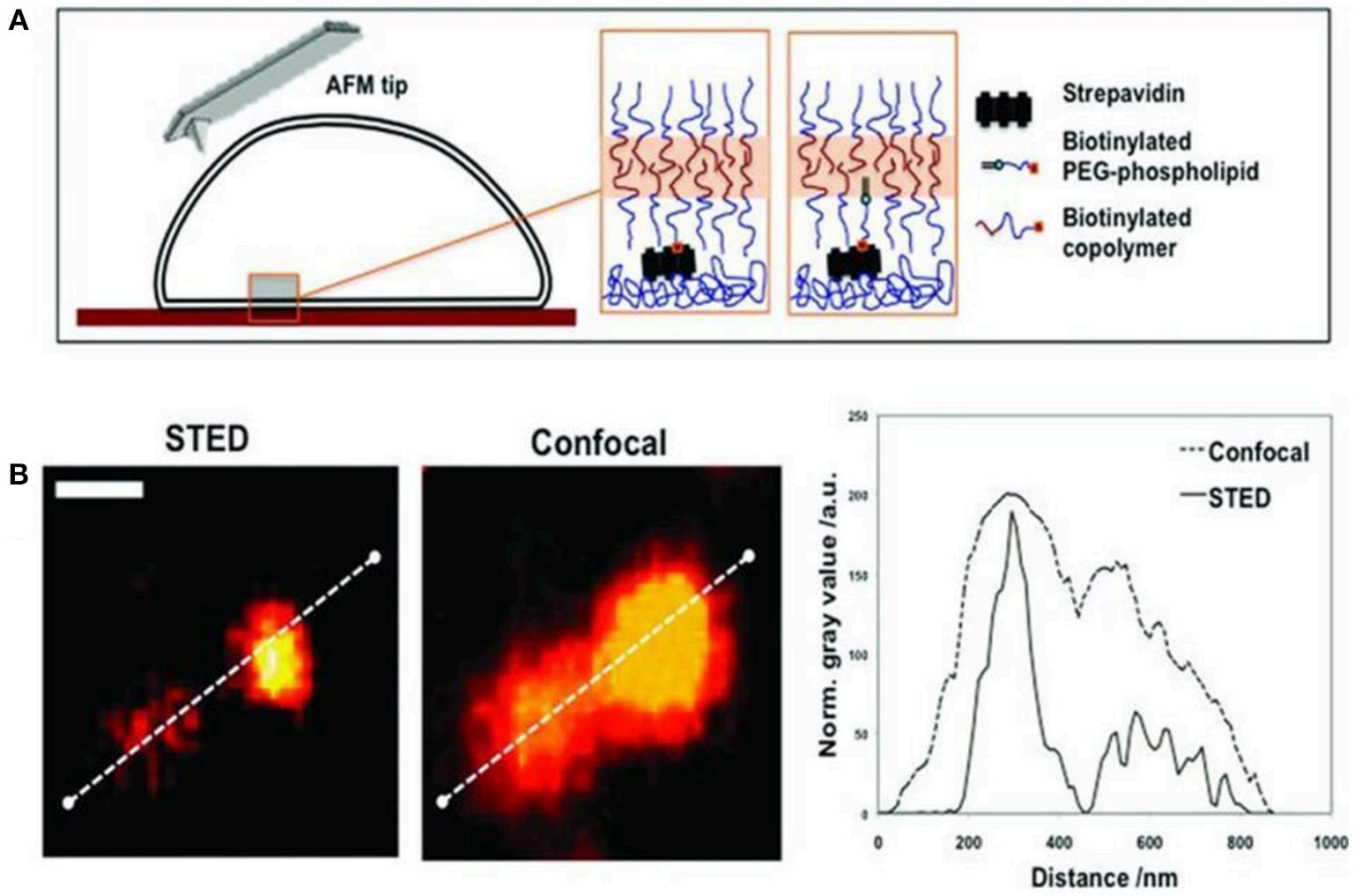
**(A)** Schematics of the biotinylated polymersome binding to a streptavidin coated surface. **(B)** STED and CSLM imaging of fluorescent (ATTO640-loaded) polymersomes immobilized by biotinylated PEG–phospholipid. Comparison of the intensity profiles confirms the enhanced resolution by STED (scale bar = 500 nm). Reproduced from Battaglia et al. (2011) with permission of Copyright © 2011 WILEY-VCH Verlag GmbH & Co. KGaA, Weinheim.

A corresponding method for polymersome immobilization on streptavidin-coated silicon wafers and petri dishes has been reported via two distinct pathways (Battaglia et al., 2011). One pathway involved blending commercially available biotin-1,2-distearoyl-sn-glycero-3-phosphoethanolamine-N-[biotinyl (polyethylene glycol)-2000] (Biotin–PEG–DSPE) with an amphiphilic block copolymer mixture to introduce the biotin to the polymersomes. The second pathway was based on the chemical functionalization of the block copolymer with biotin. In both cases, surface functionalization of poly((2-methacryloyloxy)ethyl phosphoryl choline)-*b*-poly(2-(diisopropyl-amino)ethyl methacrylate) (PMPC–PDPA) diblock copolymers with biotin was achieved (Figure 8). The surfaces were characterized at high-resolution in liquid using advanced techniques including stimulated emission depletion microscopy (STED), AFM, and force spectroscopy mapping (FSM). In particular, these methods allowed the characterization of neutral polymersomes at the nanoscale without the artifacts caused by the drying or freezing of samples. STED and AFM studies confirmed that the polymersomes can be immobilized at a surface and imaged *in situ* under native conditions. The enhanced spatial resolution offered by STED revealed that ATTO640 dye-loaded polymersomes clustered, and that the mean diameter of individual polymersomes is about half of the diameter suggested by conventional CLSM.

**Figure 9:**
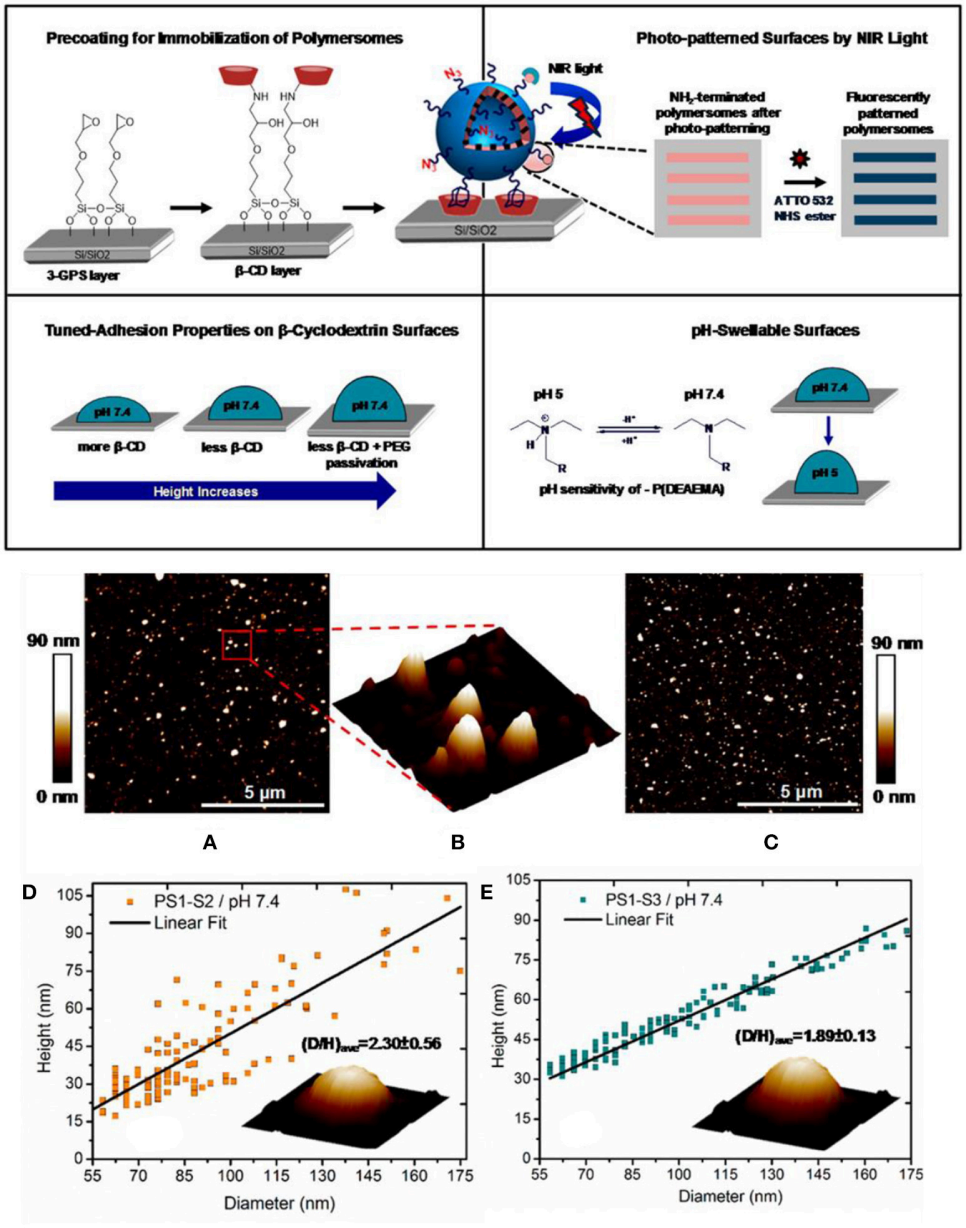
*Top*, Schematic overview of the polymersome immobilization by adamantane–β-cyclodextrin host–guest complexation and further modifications of the resulting surface. *Middle*, Peak force tapping mode liquid AFM height image of **(A)** PS1-S3 and **(C)** PS1-S2 polymersomes at pH 7.4 condition; **(B)** three-dimensional (3D) image of the marked region of the PS1-S3 polymersomes. *Bottom*, Height vs diameter relationship for **(D)** PS1-S2 and **(E)** PS1-S3 polymersomes. Reproduced from Iyisan et al. (2016) with permission of Copyright (2016) American Chemical Society.

Attachment of multifunctional, responsive, and photo cross-linked polymersomes to solid substrates was also accomplish by exploiting strong adamantane–β-cyclodextrin host–guest interactions (Figure 9) (Iyisan et al., 2016). To reduce non-specific binding and better retain spherical shape, the level of attractive forces acting on the polymersomes was tuned through both PEG passivation and a decrease of β-cyclodextrin on the corresponding substrates. In addition, polymersomes were pH responsive: they swelled under acidic conditions *in situ*. Apart from pH responsiveness, light responsiveness was achieved by modifying the polymersome surface with nitroveratryloxycarbonyl (NVOC) protected amine molecules as photo-cleavable groups prior to immobilization.

The most important techniques for the characterization of the immobilization of polymersomes on a solid support are SEM, AFM, CLSM, STED, and QCM-D. The AFM, especially AFM in liquid, can provide structural information, for example the corrugation of the modified surface, and details regarding the mechanical properties, e.g., stiffness of the polymersomes, at nanoscale resolution. SEM on the other hand, allows an overview of the system albeit the samples are dried under vacuum and require usually coating with platinum. With CLSM, one can obtain microscopy of the immobilized polymersomes or the solid support alone provided that at least one of them is fluorescent. In this case, higher resolution images recorded by STED can reveal more details than conventional fluorescence microscopy. QCM-D provides information about the amount of mass deposited on the solid substrate and simultaneously reveals conformational changes of the polymersomes upon immobilization (intact shape, rupture, strength of the attachment on the surface).

### Polymersomes Equipped With Biomolecules Turn Supports Into “Smart” Surfaces

**Figure 10:**
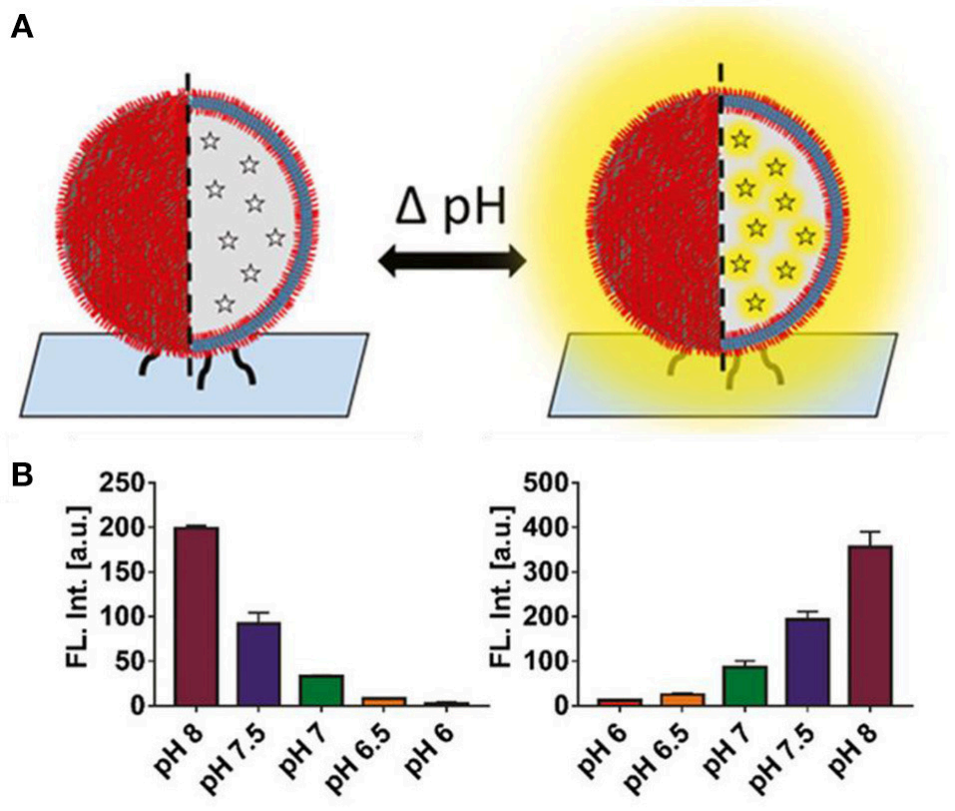
**(A)** Concept of a pH-responsive surface. Polymer nanocompartments containing a pH-sensitive dye are immobilized on a glass surface. Signaling consists of either an increase or quenching of fluorescence. **(B)** Nanocompartment pH response in solution. Decrease in fluorescent signal upon addition of lactic acid (measured pH range 8 – 6) (*right*). Increase in fluorescence upon addition of ethylenediamine, a biogenic amine (measured pH range 6 – 8) (*n* = 3) (*left*). Reproduced from Craciun et al. (2018) with permission of Copyright © 2018 Wiley-VHCA AG, Zurich, Switzerland.

Polymer-based catalytic nanocompartments on solid supports are complex systems which consist of polymersomes equipped with functional biomolecules such as encapsulated enzymes and/or proteins incorporated into the polymer membrane that have been chemically immobilized on a solid substrate to obtain specific geometries and/or additional stability (Grzelakowski et al., 2009). One example is the creation of a “smart surface” for pH reporting (Craciun et al., 2018). Using the copper-free click chemistry, a layer of polymersomes loaded with pH-sensitive dye was immobilized on a solid substrate, and reported pH changes in the external environment by a change in pyranine fluorescence (Figure 10). This concept can be widely applied to rapidly detect pH changes, and is thus of particular interest to the food industry. Another example involves polymersomes self-assembled from a mixture of non-functionalized copolymers (PMOXA_6_-*b*-PDMS_42_ -*b*-PMOXA_6_) and copolymers (PMOXA_7_ -*b*-PDMS_44_ -*b*-PMOXA_7_) functionalized with aldehyde end groups that were attached via an aldehyde-amino reaction to a glass surface that has been chemically modified with amino groups (Zhang et al., 2016). *E*. *coli* glycerol facilitator (GlpF) was functionally reconstituted into the polymersome membranes that allowed the selective diffusion of sugar alcohols to the cavity of the polymersomes, where encapsulated ribitol dehydrogenase (RDH) served as biosensing entity. This is a model of a nanosized sensor for selectively detecting sugar alcohols. Encapsulating the enzymes inside polymersomes protects them from a potentially harmful environment whilst preserving their catalytic activity.

**Figure 11:**
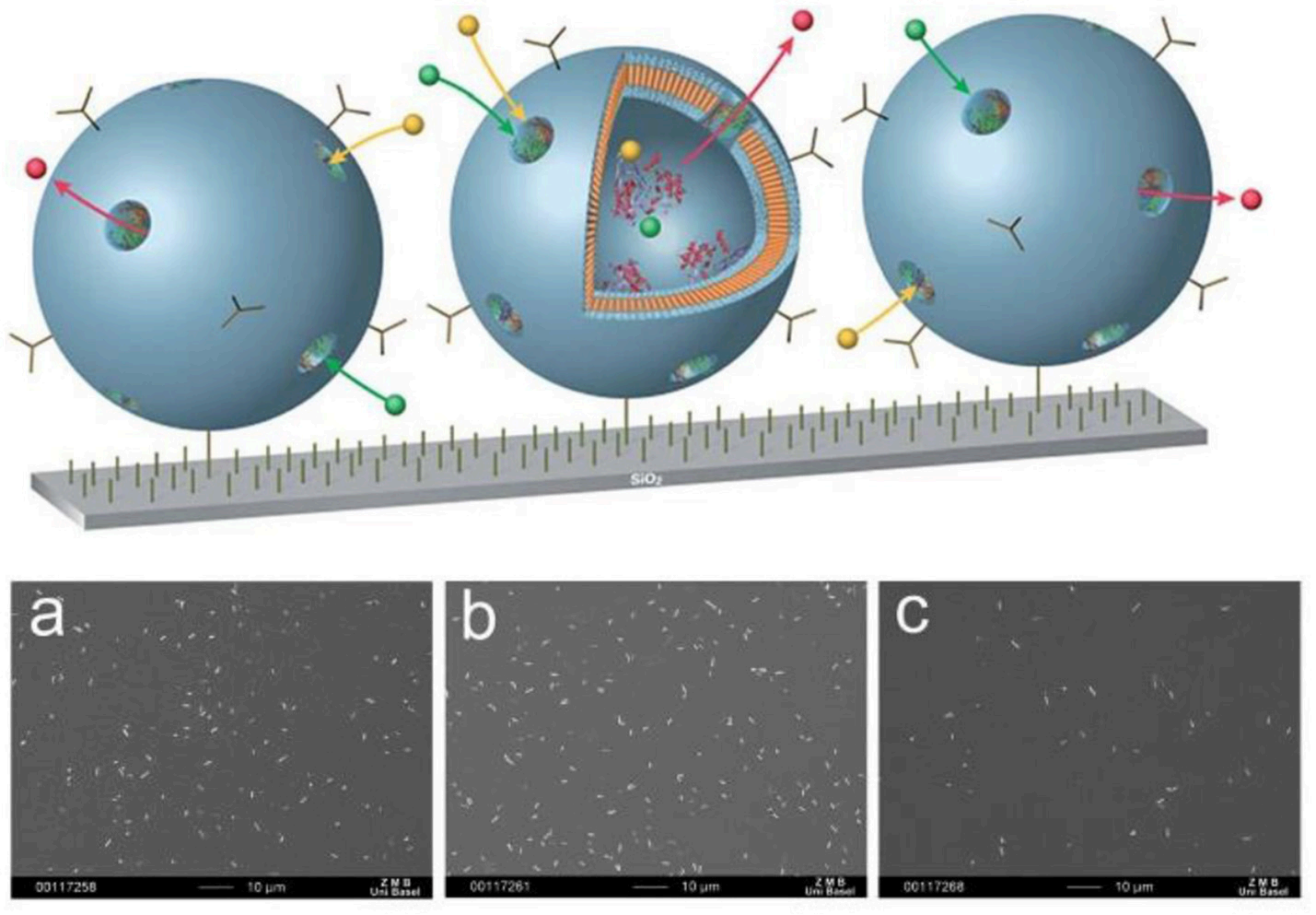
Schematic representation surface-immobilized nanoreactors producing antibiotics by encapsulated enzymes (above) SEM images of E. coli attached to: **(a)** silanized silicon surface; **(b)** surface with immobilized non-permeable polymersomes encapsulating enzyme; **(c)** surface with immobilized nanoreactors. Scale bar: 10 mm (below). Reproduced from Langowska et al. (2014) with permission with The Royal Society of Chemistry.

Moreover, surface-immobilized nanocompartments can contain an encapsulated biocatalyst, such as penicillin acylase that produces antibiotics (Figure 11) (Langowska et al., 2014). The diffusion of the substrate and antibiotics to and from the polymersomes was possible by the insertion of OmpF into the polymeric membranes. These surface-immobilized nanocompartments were enzymatically active and stable over days, producing antibiotics that efficiently inhibited bacterial growth. SEM was used to examine the growth of microorganisms that had been exposed to the surface-immobilized nanoreactors. The number of *E. coli* cells was three to four times lower on substrates with immobilized OmpF-equipped nanocompartments compared to bare silicon surfaces or to substrates with immobilized nanocompartments lacking OmpF pores.

## Supported Polymer Membranes

**Figure 12:**
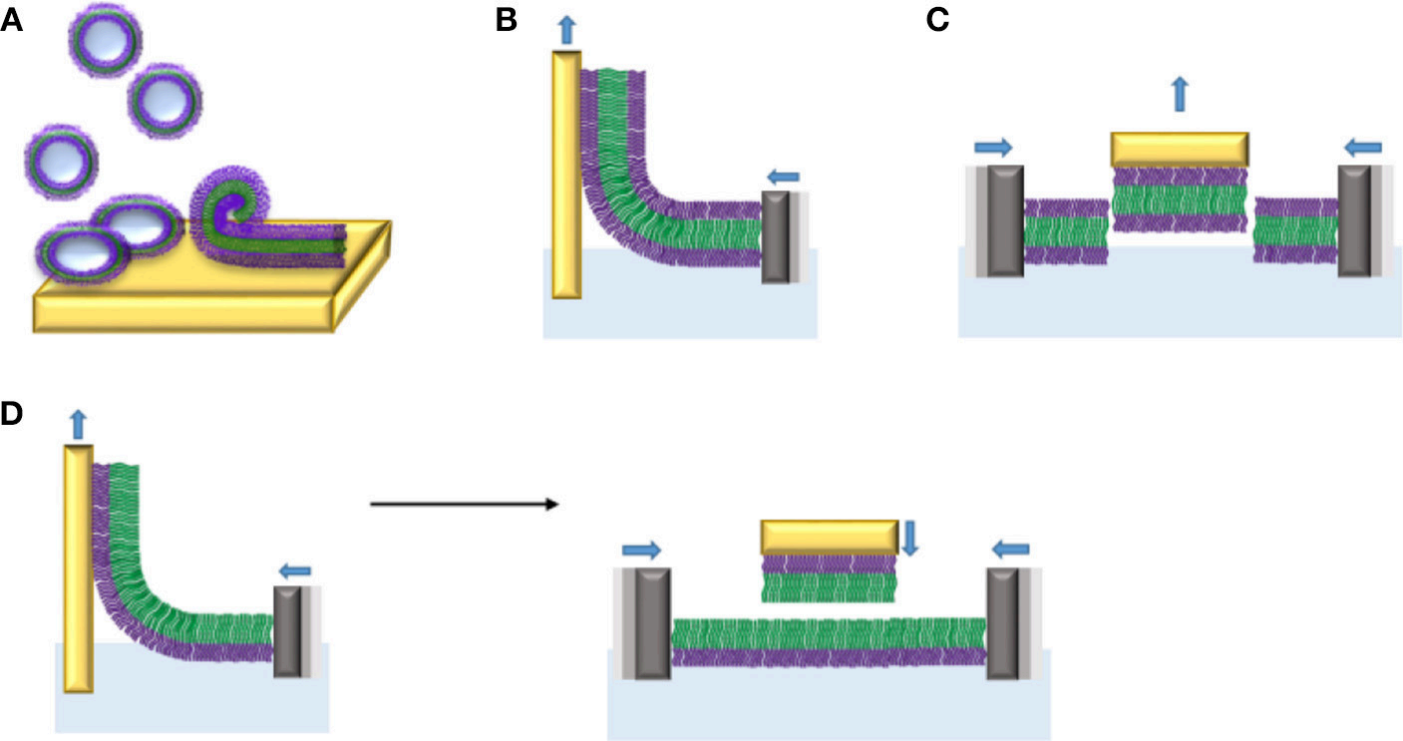
Planar membranes from amphiphilic di- and triblock copolymers on solid supports obtained by different procedures: **(A)** vesicle fusion, **(B)** monolayer transfer of triblock copolymers by Langmuir Blodgett (LB), **(C)** monolayer transfer of triblock copolymer by Langmuir Schaefer (LS), and **(D)** bilayer formation by sequential LB (left) and LS (right) transfers of monolayers made of diblock copolymers.

Amphiphilic block copolymers can form planar membranes on solid supports, so-called supported polymer membranes. The advantage of planar polymer membranes on solid supports is the possibility to use a wide range of surface sensitive characterization tools, which allows quantifying membrane associated processes based on membrane-protein and protein-protein interactions. To date, different strategies have been applied to create the polymer membranes on solid supports from different amphiphilic block copolymers (Table 1): vesicle fusion, transfer by Langmuir Blodgett (LB) and Langmuir Schaefer (LS) methods (Figure 12).

**Table 1:** Block copolymers reported to produce supported polymer membranes.

**Membrane composition**	**Method of preparation**	**Solid support**	**Protein combined**	**References**
PMOXA-b-PDMS-b-PMOXA	Vesicle fusion	Porous alumina	AqpZ	Duong et al., 2012
PDMS-b-PMOXA	LB,LS transfer	Gold, silica	MIoK1	Kowal et al., 2014
PDMAEMA-b-PBMA-b-PDMAEMA	Vesicle fusion	Mica, silica, HOPG		Rakhmatullina and Meier, 2008
PEG-b-PMCL-b-PMAEMA	LB transfer	Silica	Laccase, tyrosinase	Draghici et al., 2014, 2018
PB-*b*-PEO	LB, LS transfer	Gold	α-hemolysin	Belegrinou et al., 2011; Zhang et al., 2013

The vesicle fusion method involves a complex sequence of processes including adsorption of vesicles onto a solid support, followed by their rupture and spreading to form a continuous membrane. This method is of great interest because it is a spontaneous event based on vesicle-solid support and vesicle-vesicle interactions, which does not require any special equipment. However, to date, the vesicle fusion method has mostly been used to create supported lipid bilayers. Therefore, the role of a variety of experimental parameters such as the nature of the substrate, vesicle size, osmotic pressure, freeze-thaw pre-treatment, and lipid composition, has been studied for the formation of reproducible supported lipid bilayers (Reimhult et al., 2002, 2003; Jackman et al., 2009, 2013, 2014; Kim M. C. et al., 2016). The vesicle fusion method to produce supported polymer membranes is in its infancy and many parameters still need to be explored. It has been shown only recently that substrate charge density and hydrophobicity play a role on deformation and rupture of PBD-PEO polymersomes (Bartenstein et al., 2018). However, whether corresponding parameters have similar effects in the formation of supported block copolymer membranes by vesicle fusion remains to be elucidated. Testing the membrane properties of liposomes and polymersomes by atomic force microscopy (Jaskiewicz et al., 2012) revealed that PDMS-*b*-PMOXA exhibit an about five to seven times higher bending modulus compared to DPPC liposomes, while the Young's modulus was even lower. The higher bending modulus is related to the higher membrane thickness of PDMS-b-PMOXA polymersomes (16 nm) compared to liposomes (~5 nm). The balance between mechanical stability on one hand and bending rigidity is reflected in the different adsorption behavior on silica and mica. PDMS-*b*-PMOXA polymersomes appeared stable on silica but rupture and fuse on mica. First and foremost, the enhanced stability of polymersomes compared to lipid vesicles prevents them from rupturing on the solid support under the same conditions. Thus, rupturing conditions have to be established for each polymer. Conceivably, structural properties of resulting block copolymer membranes including electrical sealing and lateral mobility will change depending on the membrane thickness. Based on work examining polymersomes with different membrane thickness (Battaglia et al., 2006; Itel et al., 2014, 2015), it can be assumed that polymer membranes with higher thickness on a solid support have lower lateral mobility and higher electrical sealing properties. The first example of polymer membrane formation on solid support by vesicle fusion method is based on positively-charged poly(2,2-dimethylaminoethyl methacrylate)-*b*-poly(*n*-butyl methacrylate)-*b*-poly(2,2-dimethylaminoethylmethacrylate) (PDMAEMA_4_-PBMA_66_-PDMAEMA_4_) vesicles added to three different substrates, highly ordered pyrolytic graphite (HOPG), silicon oxide and mica. On mica, strong electrostatic interactions between the negatively charged mica and the positively charged polymer vesicles induced vesicle fusion that yielded a continuous polymer membrane whereas HOPG and silicon oxide caused the formation of inhomogeneous polymer membranes (Rakhmatullina and Meier, 2008). Vesicles based on poly (butadiene)-*b*-poly (ethylene oxide) (PB-*b*-PEO) diblock copolymer spread out on hydrophilic glass and ultra-smooth gold substrates at high NaCl concentrations (1.5 M) and at elevated temperature (42–45°C) (Dorn et al., 2011). A planar polymer membrane on glass was also formed by spreading hydroxyl containing polymer vesicles. However, to spread on gold surfaces, functionalization of polymer self-assemblies with sulfur contained lipoic acid (LA) groups was required. Overall, we need to improve our understanding of the interactions between polymer vesicles and different substrates in order to reliably obtain continuous and defect free polymer membranes.

Compared to the vesicle fusion method, monolayer transfers from LB and LS methods are more commonly employed to prepare solid-supported polymer membranes (Figure 12). Here, the polymer membrane is transferred to a solid support after determination of the surface pressure-area (π-A) isotherm of the monolayer which is known as “Langmuir isotherm.” The latter is obtained by compression of the polymer monolayer formed at the air-water interface until it collapses (Kita-Tokarczyk and Meier, 2008). To visualize the organization of the monolayer at the air-water interface, Brewster Angle Microscopy (BAM) was carried out in parallel. With this technique, the homogeneity of the surface layer is evaluated. After obtaining the Langmuir isotherm for a particular block copolymer, the polymer monolayer is transferred to a solid substrate by LB or LS methods (Figure 12). In LB, the substrate is vertically immersed in and removed from the aqueous subphase and the polymer membrane is transferred to the solid support during uplifting or down dipping. The LS transfer involves a horizontal and slow approach of the substrate. Both methods have the capability of producing highly ordered mono- and multi-layers, with a defined architecture and fewer defects than those obtained with the vesicle fusion method. For example, the conductive organic polymer poly(5-amino-1-naphthol) (PAN) was deposited onto hydrophilic silicon substrates by LB and LS transfer techniques (Rubinger et al., 2006). AFM characterization of the resulting monolayers revealed that monolayers produced by LS transfer were homogeneous and had fewer defects than those obtained by the LB transfer. Similarly, a solid-supported polymer membrane consisting of poly(butadiene)-*b*-poly(ethylene oxide) (PB-*b*-PEO) diblock copolymers has been produced by LB and LS transfer. First, a self-assembled monolayer of lipoic-acid terminated polybutadiene-polyethylene oxide block copolymers were immobilized on a gold support by LB transfer. Then, the solid-supported PB-*b*-PEO membrane was oriented horizontally and a second monolayer was transferred onto the first monolayer by the LS technique. This combined approach allows creating asymmetric membrane structures composed of two chemically different polymer monolayers. Surface plasmon resonance (SPR) and AFM measurements confirmed that the mono- as well as the bilayer on gold is continuous and well stable in air for about 2 h. This air stability has not been achieved with lipid membranes on solid supports since air contact immediately destroyed the lipid membranes (Holden et al., 2004; Albertorio et al., 2005). In a recent example, LB and LS transfers have been used for the deposition of triblock copolymers monolayers which lend themselves to the insertion and attachment of membrane protein (see below) (Draghici et al., 2014, 2018). Although these methods have successfully yielded a number of polymer membranes on a variety of substrates, they still have some drawbacks including the slow deposition of monolayers on the substrates and a limited resistance of the membranes to high temperatures.

Although solid supported membranes are stable and reproducible, a big disadvantage is that the membrane is in direct contact with the support, which restricts mobility. For the insertion of transmembrane proteins, the creation of space between the membrane and the solid support is of critical importance. Not only is the membrane mobility increased but also interactions of the proteins with the support, which could lead to protein denaturation, are avoided. A space of several nanometres between the membrane and substrate is usually obtained by using tethers, cushions or brushes (Sackmann and Tanaka, 2000; Smith et al., 2009; Jackman et al., 2012; Rebaud et al., 2014; Garni et al., 2017). Brushes are polymer chains that are directly attached to a surface and thus act as spacers. Brushes are obtained by the so-called “grafting—from” method, which involves the building of the polymer layer at a surface by covalent attachment of initiator molecules on the surface, followed by chain propagation through monomer addition. Another approach, called “grafting—to,” is based on the covalent attachment of preformed polymer chains on the surface. Overall, the grafting-from method is more efficient due to less steric hindrance of diffusion of the monomers in comparison to preformed, larger polymer chains. On the other hand, the preformed polymer chains used in the grafting-to method can be more easily characterized (Wang et al., 2011). However, supported membranes combined with tethers, cushions and polymer brushes are reviewed elsewhere (Sinner and Knoll, 2001; Smith et al., 2009; Zoppe et al., 2017).

A wealth of surface-sensitive characterization techniques is now available for quantitative characterization of supported model membranes and their interactions with a variety of proteins. These techniques are in general based on optical, acoustic, electrochemical or fluorescent measurement principles, which reveal the structural and functional properties of model membranes as well as the functional properties of inserted or attached proteins. One of the most important surface-sensitive techniques for polymer membranes has been ellipsometry, which can measure the optical thickness of membranes on a substrate (Richter and Brisson, 2005). More recently applied nanoscale tools include Quartz Crystal Microbalance with Dissipation monitoring (QCM-D), which enables analyzing the self-assembly of model membrane platforms in a label-free format in real-time and measuring the interactions of proteins, surfactants and cells with these surfaces in liquid (Cho et al., 2010). A direct measurement of polymer membrane thickness can be performed by AFM, which is also a useful tool to further analyze morphology, homogeneity, and structural defects of membranes. Confocal Laser Scanning Microscopy (CLSM) offers the possibility of detecting multiple fluorophores, which allows distinguishing differentially labeled membrane constituents.

### Combination of Biomolecules and Planar Membranes

Biomolecules are combined with model planar membranes to generate functional biointerfaces. The strategy of combining biomolecules with polymer membranes changes depending on the nature of the biomolecules in terms of their hydrophobic or hydrophilic characteristics. To date, the majority of biomolecules have been combined with polymeric membrane by either attaching them to the hydrophilic part membrane surface or by inserting them into the hydrophobic part of the membrane. More specifically, the biomolecules can attach to the surface of planar membranes via (i) physisorption and (ii) chemisorption with modified end-groups of the amphiphilic block copolymers forming the membranes. These two approaches principally differ with regard to the binding energy. Specifically, physisorption is based on a non-specific and reversible interaction of the biomolecules with the membrane whereas chemisorption is based on irreversible chemical binding of the biomolecules to the membrane. Upon attachment, the biomolecules may change conformation which in turn may affect their function or activity (Liu et al., 2013; Peng et al., 2014).

So far, synthetic membranes were decorated with distinct enzymes such as laccase, glucose oxidase, and horseradish peroxide to create functional surfaces (Lane et al., 2011; Draghici et al., 2014; Welch et al., 2015). For example, PEG-PMCL-PDMAEMA amphiphilic triblock copolymers were employed to create solid supported asymmetric membranes (Draghici et al., 2014, 2018). First, Langmuir isotherms of PEG-PMCL-PDMAEMA monolayers with different ratios of hydrophilic (PDMAEMA) and hydrophobic (PMCL) domains were obtained at the air-water interface. In PEG-PMCL-PDMAEMA monolayers, PEG was oriented toward the water subphase while PDMAEMA was facing the air. Then, the monolayer was transferred to a silica substrate via LB method to obtain a solid-supported membrane. Due to the different ratios of hydrophilic and hydrophobic domains, the properties of the resulting membranes differed in terms of membrane thickness, wettability, roughness and topography as assessed by ellipsometry, AFM, and contact angle measurements. Furthermore, laccase was incorporated into the polymer membrane through either immersion of the solid-supported ABC monolayer in the enzyme solution or by LB transfer of a mixed PEG-PMCL-DMAEMA amphiphilic block copolymer laccase film to a silica substrate. In both cases, laccase was stably immobilized through physical adsorption and retained its activity. More recently, PEG-PMCL-DMAEMA asymmetric monolayer and bilayer membranes were created on silica by the LB method (Draghici et al., 2014). Then, laccase and tyrosinase were adsorbed to the membranes to generate functional surfaces for phenol sensing. Depending on the membrane properties, the level of enzymatic activity changed. In addition, PEG-PMCL-DMAEMA bilayer membranes provided enhanced stability and enzymatic activity compared to monolayer membranes.

**Figure 13:**
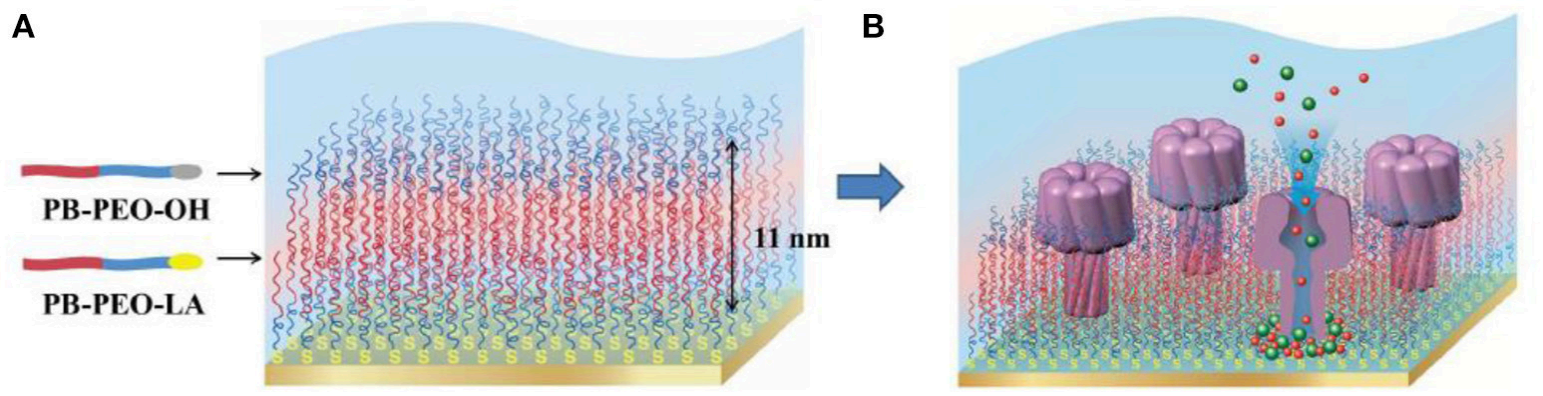
Schematic representation **(A)** PB-PEO-OH and PB-PEO-LA diblock copolymers were transferred onto gold substrates by LB-LS technique to form a polymer TSSBM, which is suitable for α-hemolysin insertion **(B)**. Reproduced from Zhang et al. (2013) with permission of https://creativecommons.org/licenses/by-nc-nd/3.0/.

Membrane proteins can be inserted into polymer membranes either during the membrane formation process or after the membrane is formed. For example, alpha hemolysin has been successfully inserted into supported polymer membranes made of PB-*b*-PEO diblock copolymers (Figure 13) (Zhang et al., 2013). The insertion of alpha hemolysin into the membrane was mediated by voltage destabilization. This approach allowed a permanent and functional insertion of alpha hemolysin, as confirmed by a flow of ions across the membrane. However, specific conditions are required including (i) a homogenous and stable polymer membrane, (ii) sufficient membrane fluidity to host the proteins, (iii) a spacer between membrane and solid support to create a reservoir for ion flux and to inhibit substrate interactions of the protein that might lead to denaturation.

**Figure 14:**
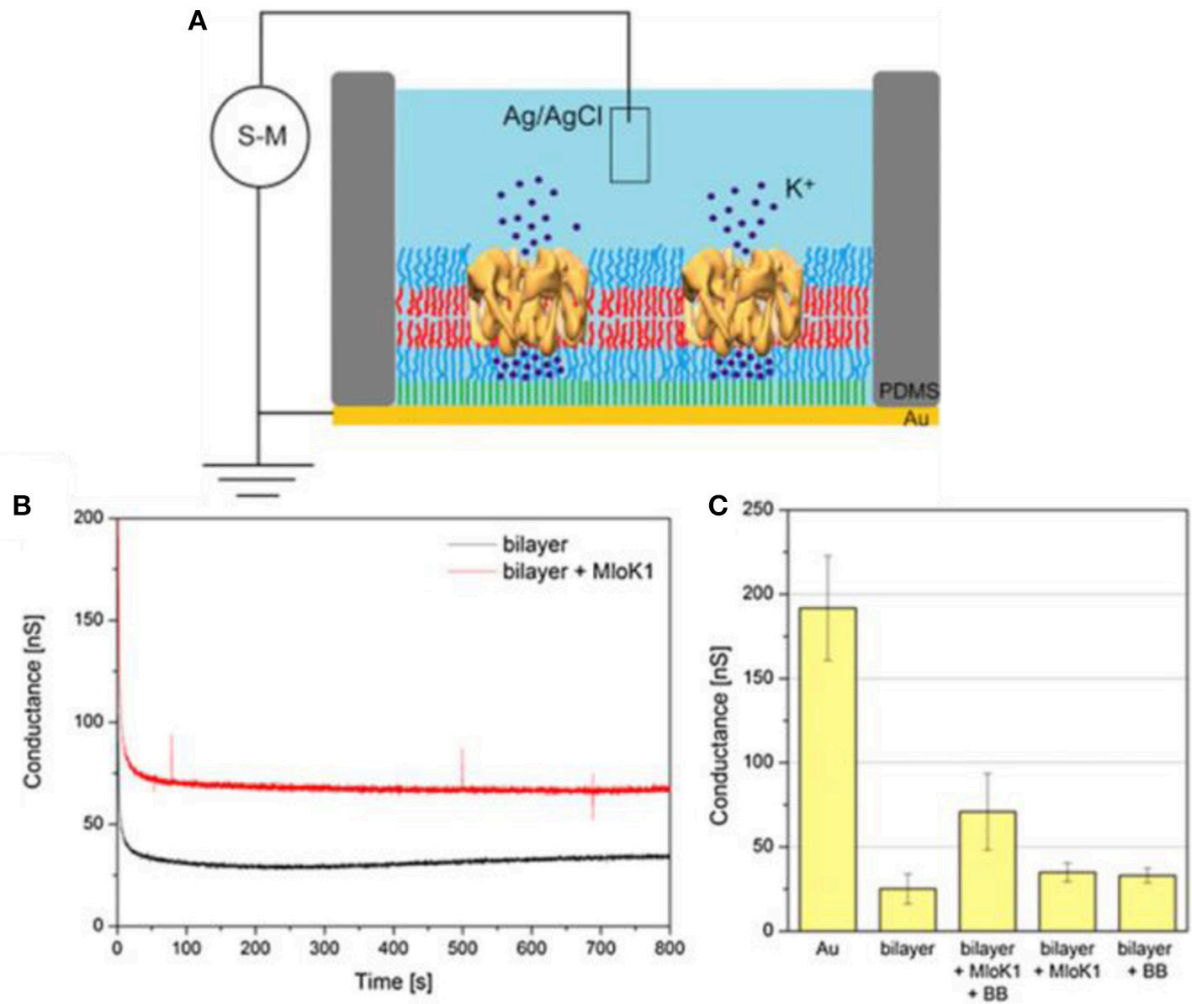
**(A)** Schematic representation of the setup used for conductance measurements through polymer membranes (S-M – source-meter, PDMS – poly(2-methyl-2-oxazoline) stamp). **(B)** Time course for conductance of solid-supported polymer bilayer (black line) and solid-supported polymer bilayer with incorporated MloK1 (red line). **(C)** Conductance measured at a constant applied voltage of 40 mV (Au – gold substrate, BB – Bio-Beads). Reproduced from Kowal et al. (2014) with permission of Copyright © 2014 Elsevier Ltd.

Biobeads mediated membrane protein insertion has been also applied for protein insertion into supported membranes (Kowal et al., 2014). More specifically, PDMS-*b*-PMOXA diblock copolymers were used to create membranes on silica and gold surfaces using both LB and LS techniques. To achieve functional insertion of the membrane protein MloK1, a cyclic nucleotide-modulated potassium channel from *Mesorhizobium loti*, both the protein and the polymer membrane were destabilized by biobeads. In this particular study, the biobeads provided the driving force for the insertion of the membrane protein into the polymer membrane. The functionality of inserted proteins was investigated by measuring the electrical conductance (Figure 14). The conductance increased only when protein incorporation into the membrane was carried out in the presence of bio-beads. Another method to prepare planar protein-polymer hybrid membranes is by rupturing polymersomes equipped with membrane proteins with the aid of covalent bonding of the polymer to gold substrates (Duong et al., 2012). For example, Aquaporin Z (Aqp Z) transmembrane water channels were first incorporated into disulfide functionalized PMOXA-*b*-PDMS-*b*-PMOXA polymersomes. Then, polymersomes with and without aquaporin-Z induced to spread on gold-coated solid substrates by the covalent bonding of disulfide groups to the gold, yielding a highly permeable membrane that allows passive diffusion of small solutes such as ions, nutrients or antibiotics.

## Present and Future Perspectives on Biomedical Applications

The combination of biomolecules with block copolymers turns the nanoassemblies reviewed in this article into prime candidates for a broad range of biomedical applications, notably as biosensors (Islam et al., 2014; Sun et al., 2015; Idrissi et al., 2018), in diagnostic imaging (Choi et al., 2012; Mi et al., 2017; Quader and Kataoka, 2017), as drug/gene delivery systems, mainly in the context of cancer treatment (Nishiyama et al., 2016; Quader and Kataoka, 2017; Varela-Moreira et al., 2017; Cabral et al., 2018; Cheng et al., 2018; Mukerabigwi et al., 2018; Wang et al., 2018a; Wang J. et al., 2018), in the treatment of infectious disease (Aderibigbe, 2017; Liu et al., 2017) and the fight against biofilms (Liu et al., 2018), and in regenerative medicine (Torabinejad et al., 2014; Mota et al., 2015; Raisin et al., 2016; Susanna et al., 2017; Rey-Rico and Cucchiarini, 2018).

Block copolymers, if appropriately selected are well-suited to fulfill key requirements of biomedical applications including biocompatibility, stability, controllable size, robustness and tunable surface chemistry. For *in vivo* applications, in particular therapeutic applications, block copolymer-based nanomedicines should stably circulate in the vascular system while avoiding unspecific interactions with blood components, specifically extravasate at the site of disease, followed by an efficient uptake and eventually target-specific release of the cargo. Ideally, the design of the nanovehicles should entail controllable drug release, increase of drug bioavailability, and reduction of adverse effects.

Other appealing features of polymer nanoassemblies include the ability to host a large range of molecules. Either in their cavity if cargoes are hydrophilic e.g., small molecule drugs (hydrophilic doxorubicin, DOX•HCL) (Simon-Gracia et al., 2016), proteins (Wang L. G. et al., 2012), nucleic acids (Lomas et al., 2011; Kim H. J. et al., 2016), and imaging agents (Canton and Battaglia, 2013), and within polymersome membranes (e.g., paclitaxel; doxorubicin base, DOX; Xu et al., 2014) and polymer micelles if the compounds are hydrophobic (Anraku et al., 2010; Simon-Gracia et al., 2016). A real advantage of the polymersome architecture is the possibility of a simultaneous loading with hydrophilic and hydrophobic molecules.

As the ability to respond to environmental conditions is crucial for many biomedical applications, considerable efforts have been directed to the development of “smart” polymer nanoassemblies that respond to stimuli (physical, such as pH, temperature, light, magnetic field, or chemical, as for example the presence of specific molecules). Stimuli-sensitive polymersomes have emerged as delivery systems where the release of the encapsulated contents can be modulated by the stimulus (Alsuraifi et al., 2018; Kalhapure and Renukuntla, 2018; Rao et al., 2018; Wang et al., 2018a). Conceivably, stimuli-responsive release may result in significantly enhanced therapeutic efficacy and minimized side effects in clinical applications (Alsuraifi et al., 2018; Yang et al., 2018).

The past two decades have seen a boost in the development of polymer based drug and gene delivery systems and their rapid increase in in cancer diagnosis and treatment (Duncan, 2003; Biswas et al., 2016; Varela-Moreira et al., 2017; Zhao et al., 2017; Cabral et al., 2018). Both tasks can be combined into one modality by dual-use “theranostic” nanocarriers engineered to simultaneously deliver therapeutic and imaging cargoes (Nishiyama et al., 2016; Quader and Kataoka, 2017; Cabral et al., 2018). A variety of micellar and vesicular nanocarriers have been designed to deliver nucleic acids to specific sites *in vitro* and *in vivo* (Raisin et al., 2016, 2017). Specifically, multifunctional polymer gene vectors are highly promising for siRNA delivery to tumor cells and tissues (Sun et al., 2012; Du et al., 2017; Lee et al., 2017). Recent examples include polymeric micelles for the co-delivery of siRNA and hydrophobic drugs (Lee et al., 2017), and a multifunctional siRNA delivery system with high gene silencing efficiency and antimicrobial ability (Zhou et al., 2018). Similarly, polymersomes that transformed to polymeric micelles at acidic pH also showed successful gene transfection ability as nonviral vectors in human cell lines (Laskar et al., 2017).

In the following we will first address the main biomedical applications of polymeric micelles before pointing out where polymersome characteristics broaden these applications. Finally, we will look into how planar supported polymer-biomolecule assemblies are used as biosensing platforms.

Polymeric micelles (PMs) have become one of the most promising nano-delivery systems for the treatment of cancers (Nishiyama et al., 2016; Cabral et al., 2018; Wang J. et al., 2018). Their size of below 100 nm enables them to effectively overcome biological barriers. PMs have been used for the delivery of many different cargoes, including conventional chemotherapeutic drugs, photosensitizers, immunomodulators, and biological macromolecules, such as proteins and genes. In particular, PEG-PLA micelles have been intensely studied because their PEG shell prevents the unspecific adsorption of plasma proteins and the interactions with phagocytes while the PLA core can effectively encapsulate a variety of therapeutics. Furthermore, biocompatible poly(ethylene oxide)-poly(propylene oxide)-poly(ethylene oxide) (PEO-PPO-PEO) triblock co-polymers have been used as carriers for the IV injection of the anti-inflammatory agent (methylprednisolone) (Chen et al., 2008) and DOX (Gao et al., 2005), as well as oral non-viral gene delivery systems (Chang et al., 2004), intramuscular sustained release formulations (Wenzel et al., 2002), and trans-dermal patch or inhalation applications (Liaw and Lin, 2000; Chao et al., 2007).

By now, several polymeric micelles are in clinical trials, predominantly in cancer therapy, with the aim to achieve better patient outcome based on the advantages offered by block copolymers (Thakor and Gambhir, 2013; Kim et al., 2014; Kim H. J. et al., 2016; Nishiyama et al., 2016; Mukai et al., 2017; Park et al., 2017; Rao et al., 2018).

Among the limitations of polymeric micelles in clinical applications, poor micelle stability and/or insufficient drug retention, both of which lower drug targeting efficiency, are most critical. The dilution of polymeric micelles after intravenous injection and/or interactions between polymer building blocks and blood components reduce the blood circulation of polymeric micelles by promoting micelle dissociation or enhanced clearance. For example, the adsorption of opsonins present in the plasma to the surface of polymeric nanostructures affects their *in vivo* stability and leads to their early removal from the circulation. So far, little work has been done on the influence of PM core chemistry on protein adsorption (Toncheva et al., 2003; Garg et al., 2015). Moreover, insufficiently shielded drug loads also interact with blood components (e.g., plasma proteins, cells) during circulation which leads to rapid clearance or off-target effects.

The nature of the hydrophilic shell plays a crucial role in the protection of the core. For a variety of biomedical applications, formulations with poly (ethylene glycol) (PEG) as the hydrophilic block have been the polymer of choice. With the graft copolymer, poly (D,L -lactide-co−2-methyl-2-carboxytrimethylenecarbonate)-g-poly(ethylene glycol) (P(LA-co -TMCC)-g -PEG), it was shown that increasing the PEG density improves PM stability of the resulting self-assembled micelle. Amongst the many methods that have been studied to control protein adsorption to PMs, coating the nanoparticle surface with polyethylene glycol (PEGylation) is the most prominent and well-documented in the literature (Lu et al., 2011; Mishra et al., 2016; Suk et al., 2016). A further advantage of PEGylation is that PEG is widely accepted to be a non-toxic, non-immunogenic polymer, although evidence to the contrary exists (Garay and Labaune, 2011; Schellekens et al., 2013). Similarly, poly (ethylene oxide)-based hydrophilic shells were shown to have little protein adsorption (Garg et al., 2015). However, protein adsorption increased with increase of the hydrophobicity and molecular weight of the core-forming block.

To enhance PM stability, strategies involving physico-chemical modifications of polymers are also intensely pursued (Jiang et al., 2007; Shi et al., 2017). For example, π-π stacking, stereocomplexation, hydrogen bonding, host-guest complexation, free radical polymerization, click chemistry, disulfide and hydrazone bonding have improved the stability of polymeric micelles. In particular, covalent cross-linking of polymeric core and shell and the introduction of electrostatic interactions have contributed to PM stabilization.

The *in vivo* fate of PM delivery systems is also governed by the ability to create an immune response. Polymeric micelles are widely considered non-immunogenic although studies directly addressing the immunogenicity of micelles assembled from amphiphilic block copolymers are scarce (Shiraishi et al., 2016). Immunogenicity of different PEO-*b*-PCL micelles measured in terms of promoting the phenotype maturation and cytokine secretion by dendritic cells showed that irrespective of the micellar core structure, all tested micelles were non-immunogenic in bone marrow-derived dendritic cells (Garg et al., 2015).

Nevertheless, drug loading efficiency, prevention of rapid clearance, maintenance of the nanocarrier integrity in the circulation, and the controlled disassembly for drug release at specific target sites remain challenges that need to be met by engineering next generation polymers.

Besides primary passive targeting through the enhanced permeability and retention (EPR) effect, polymeric micelles have been propitious for site-specific cargo delivery either through adjustment of their properties in response to particular local stimuli (such as the slight increase in temperature or acidity in the tumor microenvironment) and/or by active targeting through conjugation to targeting moieties (ligands, antibodies), which may modulate the activity of the loaded drugs at the targeted sites, even at the subcellular level (Yao et al., 2017; Yi et al., 2018). Moreover, recent applications also show a clear trend toward the utilization of multiple responsive micelles with more than one type of therapeutic payload (Deng et al., 2018; Wang J. et al., 2018).

A distinct advantage of polymersomes over micelles (both polymer and lipid) is their large watery cavity which is ideal for loading and shielding water-soluble agents (Alibolandi et al., 2017). This is of particular interest as many proteins (e.g., antibodies, Herceptin, IFN-γ) have striking anticancer activities and, when formulated with properly designed nanovehicles, are emerging as novel nanomedicines in cancer therapy (Cheng et al., 2018). Strategies to overcome the low protein loading efficiency often associated with common polymers are being intensely pursued. In addition, encapsulation of hydrophilic biomolecules protects them from proteolytic attack and thereby increases their life-time in the blood circulation. The presence of a surface-exposed hydrophilic PEG domain of polymersomes is known to decrease immune reactivity (stealthiness), which also helps to prolong circulation time.

Moreover, the versatility of membrane thickness and permeability render polymersomes promising candidates for drug delivery and biosensing, especially when membrane engineering confers stimuli-responsiveness upon the vesicles which further adds to the controlled release of its cargo (Lee and Feijen, 2012). Last but not least, polymersomes can be armed with targeting moieties that aid in tumor specificity. Functionalized polymersomes have been used for theranostic purposes in animal models for different diseases such as cancers, inflammations and brain-related disorders (Sarkar and Paira, 2018; Wang et al., 2018b).

A preclinical study in a murine colon cancer model using doxorubicin encapsulated in PEG-PLGA polymersomes has shown that the polymersomal formulation of DOX is as therapeutically effective as the clinically approved liposome based drug carrier Doxil-mimic but at a lower administrated dose (Alibolandi et al., 2017). The polymersomal formulation of DOX can potentially limit off-site effects of Doxil due to its biodegradability and sustained release properties without compromising on the safety features.

However, despite the improved structural properties of polymersomes, the FDA has so far not approved their clinical application. Limitations that need to be overcome for drug delivery purposes include insufficient knowledge on biocompatibility, biodistribution, and pharmacokinetics of polymersomes. To advance the clinical application of polymersomal- protein nano carriers, future efforts will have to aim at improving the biosafety and the control of production of polymersomes, and at optimizing tumor specificity.

By combining polymersomes with corresponding biomolecules they can be turned into ideal therapeutic catalytic nanocompartments (CNCs) with a confined reaction space where the loaded fragile catalysts are protected, that can be targeted to a specific site in the body where they are selectively activated. Similarly, CNCs have been designed that have antioxidant characteristics (Axthelm et al., 2008) or even act as artificial organelles inside cells, e.g., in the detoxification of reactive oxygen species (Tanner et al., 2013; Einfalt et al., 2018). Therapeutic CNCs which can efficiently produce highly toxic anticancer drugs *in situ* from low-toxic prodrugs or some biomolecules in tumor tissues have recently been proposed as a novel nanoplatform to treat diseases (Mukerabigwi et al., 2018). The *in situ* production of therapeutic agents can maximize the therapeutic efficacy while lowering systemic toxicity at the same time.

In light of the global increase of multidrug-resistance, the biomedical application of polymeric nanocarriers delivering antimicrobials is rapidly gaining significance (Liu et al., 2017, 2018; Lanzilotto et al., 2018). Recent studies have shown anti-infectious activity of PMs in murine *in vivo* models (Liu et al., 2018; Zhou et al., 2018) and the eradication of biofilms in human *ex vivo* models (Liu et al., 2018).

Polymer-based membranes are emerging technologies in drug screening and development as illustrated by G protein-coupled receptors (GPCRs). Recently, the functional insertion of the GPCR proteorhodopsin in polymersomes has been reported (Goers et al., 2018), indicating the potential of polymer membranes to capture GPCRs and to serve as a drug screening platform.

Biosensors are important in diagnostics because their sensitivity can be higher compared to normal sensors due to the specificity of the biomolecules that serve as active elements. Polymer membrane based biosensors have been applied in fluids, as detecting surfaces, and in the form of immobilized nanoreactors on functionalized surfaces (Turner, 2013). For example, micelles of poly (n-butylmethacrylate)-block-poly (N,N-dimethylaminoethyl methacrylate) (PnBMA-*b*-PDMAEMA), and choline oxidase were used to obtain bilayer films on conductive surfaces at different pH-values (Sigolaeva et al., 2014). Sequential electrostatic adsorption of diblock copolymer micelles combined with the additional possibility of crosslinking enzymes within such films produced highly active and stable biosensor coatings. Self-assembled polymer layers with an immobilized enzyme placed on an electrode is an established approach for fabricating implantable biosensors in medical diagnostics (Rothwell et al., 2010). Examples include glucose sensing in the context of diabetes (Zhang et al., 2010) and the detection of uric acid in the diagnosis and treatment of hyperuricemia and gout (Spieker et al., 2002). Another example indicates that porous polystyrene-*b*-poly(4-vinyl pyridine) (PS-*b*-P4VP) block copolymer films are a good matrix for the immobilization of glucose oxidase (Guo T. et al., 2018). The enzyme film retained its native structure and bioactivity in PS-*b*-P4VP films, and the direct electron transfer between the enzyme and the electrode was enhanced compared to other glucose biosensors.

The plethora of biomolecules that can be inserted in, or attached to polymer membranes enables the rational design of biointerfaces as substrates for cells and tissues. To date, studies exploiting this application are limited (Zhang et al., 2012; Sterner et al., 2014; Kilicay et al., 2016). The design of new types of polymer-based biointerfaces, for example antimicrobial coatings from hybrid polymer micelles (Galvão et al., 2018) or planar membranes with anti-microbial properties as coatings for medical devices, will hopefully provide innovative preventive and therapeutic measures that open new avenues in regenerative medicine.

Despite continuing challenges of incorporating and observing membrane proteins in polymer membranes, the ability to harness polymeric membranes improves. It is likely that the future generations of model polymer membranes will involve more complex assembly compositions (proteins and lipids) and combinations (networks), leading to insights into essential biological processes that help us understand and fight pathological conditions. Future developments in block copolymer assemblies are expected to improve and expand biomedical applications and significantly advance the treatment of cancer and many other diseases.

## Author Contributions

SYA, MK, SD, and C-AS did literature research and wrote the manuscript. WM and CP provided comments and helped in finalizing the manuscript. All authors reviewed the final version of the manuscript and approved it for publication.

### Conflict of Interest Statement

The authors declare that the research was conducted in the absence of any commercial or financial relationships that could be construed as a potential conflict of interest.

## Abbreviations

a_o_: the contact area of head group
ATRP: (atom transfer radical polymerization(diisopropyl-amino) ethyl methacrylate)
3-caprolactone
4F3 NB: 4-fluoro-3-nitrobenzoic acid
4FB: 4-foramybenzoate
ABTS, 2: 2′-azinobis (3-eth-ylbenzothiazoline-6-sulfonic acid)
AFM: atomic force microscopy
ATOTA-COOH 2-(N-methyl-N-2-4-carboxyl butyl amino) (6, 10-bis(N: N-dimethylamino)) trioxatriangulenium chloride
b-anti-ICAM-1: biotinylated-anti-ICAM-1
BAM: Brewster Angle Microscopy
Biotin–PEG–DSPE: biotin-1,2-distearoyl-sn-glycero-3-phosphoethanolamine-N-[biotinyl] (polyethylene glycol)
CalB: Candida antarctica lipase B
CGRGS: Thiol-containing targeting peptides
CLSM: confocal laser scanning microscopy
Con A: concanavalin A
CMC: critical micellar concentration
CNC: catalytic nanocompartments
cRGD: cyclic arginine-glycine-aspartic acid
Cryo-SEM: Cryo-scanning electron microscopy
CuAAC: Cu(I)-catalyzedazide–alkyne cycloaddition
DACHPt: (1, 2-diaminocyclohexane)platinum
DBCO: dibenzocyclooctyne
DLS: dynamic light scattering
DoE: Design of experiments
DOPC: 1, 2-dioleoyl-sn-glycero-3-phosphocholine
DOX: doxorubicin
DOX•HCL: hydrophilic doxorubicin
FSM: force spectroscopy mapping
G1P: glucose-1-phosphate
G6P: glucose-6-phosphate
gA: Gramicidin A
GlpF: *E. coli* glycerol facilitator
Gox: glucose oxidase
GPCRs: G protein-coupled receptors
GUVs: giant unilamellar vesicles
HRP: horseradish peroxidase
HyNic: 6-hydrazinocotinate acetone hydrazine
LA: lipoic acid
LB: Langmuir Blodgett
l_c_: length of hydrophobic block
LS: Langmuir Schaefer
LCST: lower critical solution temperature
m: number of monomer type
MDOX: doxorubicin-loaded micelles
MEO_2_MA 2-(2′−methoxyethoxy)ethyl methacrylate
Mn: number average molecular weight
Mw: weight average molecular weight
n: number of monomer
NADPH: Nicotinamide adenine dinucleotide
N_j_: degree of polymerization of each monomer
NMP: nitroxide mediated polymerization
NTA: nitrilotriacetic acid
NVOC: nitroveratryloxycarbonyl
OEGMA: oligo(ethylene glycol) methacrylate
Omp: outer membrane proteins
OmpF: outer membrane proteins F
OX26: Mouse-anti-rat monoclonal antibody
P: packing parameter
PAN: polyacrylonitrile
PAN: poly (5-amino-1-naphthol)
PB-PEO: polybutadiene-poly(ethylene oxide)
PBD-b-PEO: poly(butadiene)-b-poly(ethylene oxide)
PDMAEMA-PBMA-PDMAEMA: poly(2,2-dimethylaminoethyl methacrylate)-block-poly(n-butyl methacrylate)-block- poly(2,2-dimethylaminoethyl methacrylate)
PDMS: polydimethylsiloxane
PEE-b-PEO: poly(butadiene)-b-poly(ethylene oxide)
PEG: polyethylene glycol
PEG-PE: polyethylene glycol-phosphatidyl ethanolamine
PEO-b-PCL: poly(ethylene oxide)-block-polycaprolactone
PEO-b-PCL-b-PMOXA: poly(2-methyloxazoline)-b-poly(dimethylsiloxane)-b-poly(2-methyloxazoline)
PEO-PPO-PEO poly(ethylene oxide)-poly(propylene oxide)-poly(ethylene oxide)
PEG-PDLLA: Poly(ethylene glycol)-*b*-poly(D,L-lactide)
PFS: polyferrocenylsilane
PFS-PDMS: polyferrocenylsilane- polydimethylsiloxane
PFS-PI: polyferrocenylsilane- polyisoprene
PFS-PMVS: polyferrocenylsilane- polymethylvinylsiloxane
PGM: phosphoglucomutase
PI: polyisoprene
PICsomes: polyion complex vesicles
PID118-PLA71: poly(N-isopropylacrylmide-co-N, N'-dimethylacryl-amide) 118 -b-poly(D,Llactide)71
PK: phosphoglycerate kinase
P(LA-co -TMCC)-g –PEG poly (D,L -lactide-co−2-methyl-2-carboxy trimethylene carbonate)-g-poly(ethylene glycol)
PM: polymer micelles
PMAA: poly (methacrylate acid)
PMPC–PDPA poly((2-methacryloyloxy)ethyl phosphorylcholine)- block-poly(2-(diisopropyl-amino)ethyl methacrylate) p(MEO_2_MA-OEGMA) poly (2-(2′−methoxyethoxy)ethyl methacrylate)-poly (oligo(ethylene glycol) methacrylate)
PMVS: polymethylvinylsiloxane
PNIPAM: poly(N-isopropyl acrylamide)
PPO: poly(propylene oxide)
PR: proteorhodopsin
PS-b-PIAT: polystyrene-b-poly (l-isocyanoalanine) (2-thio-phen-3-yl-ethyl)
PS-PAA: polystyrene-poly (acrylic acid)
QCM: quartz crystal microbalance
Rg: radius of gyration
Rh: hydrodynamic radius
RAFT: reversible addition-fragmentation chain transfer
RCA1: *Ricinus communis* agglutinin 1
RDH: ribitol dehydrogenase
SEM: scanning electron microscopy
SLS: static light scattering
SPAAC: strain-promoted azide–alkyne cycloaaddition
SPR: surface plasmon resonance
STED: stimulated emission depletion microscopy
TAT: transcriptional transactivator
TCEP: Tris(2-carboxyethyl)phosphine hydrochloride
TEM: transmission electron microscopy
UCST: upper critical solution temperature
V: the volume of the hydrophobic block
VS-PEO-b-PMCL: vinyl sulfone functionalized- poly (ethylene oxide)-block-poly (g-methyl- caprolactone)
X_ij_ the associated interaction parameters αHL: alpha-hemolysin
*Đ*: dispersity.
